# Protein recoding by ADAR1-mediated RNA editing is not essential for normal development and homeostasis

**DOI:** 10.1186/s13059-017-1301-4

**Published:** 2017-09-05

**Authors:** Jacki E. Heraud-Farlow, Alistair M. Chalk, Sandra E. Linder, Qin Li, Scott Taylor, Joshua M. White, Lokman Pang, Brian J. Liddicoat, Ankita Gupte, Jin Billy Li, Carl R. Walkley

**Affiliations:** 1 St. Vincent’s Institute of Medical Research, 9 Princes St, Fitzroy, 3065 VIC Australia; 2 Department of Medicine, St. Vincent’s Hospital, University of Melbourne, Fitzroy, VIC 3065 Australia; 3 Department of Genetics, Stanford University, Stanford, CA 94305 USA

**Keywords:** ADAR1, RNA editing, MDA5, Development, dsRNA, Innate immunity, Epitranscriptome

## Abstract

**Background:**

Adenosine-to-inosine (A-to-I) editing of dsRNA by ADAR proteins is a pervasive epitranscriptome feature. Tens of thousands of A-to-I editing events are defined in the mouse, yet the functional impact of most is unknown. Editing causing protein recoding is the essential function of ADAR2, but an essential role for recoding by ADAR1 has not been demonstrated. ADAR1 has been proposed to have editing-dependent and editing-independent functions. The relative contribution of these in vivo has not been clearly defined. A critical function of ADAR1 is editing of endogenous RNA to prevent activation of the dsRNA sensor MDA5 (*Ifih1*). Outside of this, how ADAR1 editing contributes to normal development and homeostasis is uncertain.

**Results:**

We describe the consequences of ADAR1 editing deficiency on murine homeostasis. *Adar1* ^*E861A/E861A*^ *Ifih1* ^*-/-*^ mice are strikingly normal, including their lifespan. There is a mild, non-pathogenic innate immune activation signature in the *Adar1* ^*E861A/E861A*^ *Ifih1* ^*-/-*^ mice. Assessing A-to-I editing across adult tissues demonstrates that outside of the brain, ADAR1 performs the majority of editing and that ADAR2 cannot compensate in its absence. Direct comparison of the *Adar1* ^*-/-*^ and *Adar1* ^*E861A/E861A*^ alleles demonstrates a high degree of concordance on both *Ifih1* ^*+/+*^ and *Ifih1* ^*-/-*^ backgrounds, suggesting no substantial contribution from ADAR1 editing-independent functions.

**Conclusions:**

These analyses demonstrate that the lifetime absence of ADAR1-editing is well tolerated in the absence of MDA5. We conclude that protein recoding arising from ADAR1-mediated editing is not essential for organismal homeostasis. Additionally, the phenotypes associated with loss of ADAR1 are the result of RNA editing and MDA5-dependent functions.

**Electronic supplementary material:**

The online version of this article (doi:10.1186/s13059-017-1301-4) contains supplementary material, which is available to authorized users.

## Background

It is now established that direct RNA modifications, collectively referred to as the epitranscriptome, are widespread, evolutionarily conserved, and contribute significantly to the complexity of gene regulation [1, 2]. A highly prevalent modification is the deamination of adenosine to inosine (A-to-I editing) in double-stranded RNA (dsRNA). A-to-I editing is catalyzed by adenosine deaminase acting on RNA (ADAR) proteins. It is estimated that there are over 100 million potential A-to-I editing sites in humans and tens of thousands in mice [3, 4], with the majority residing within repetitive sequences, such as short interspersed nuclear elements (SINEs), *Alus* (primates only), and long tandem repeats (LTRs) [5]. Depending on the sequence context—coding sequence, intronic region, untranslated regions, non-coding RNAs—editing can have varying consequences for gene expression [6]. Outside of a small number of examples, the cellular and organismal consequences of A-to-I editing are relatively poorly understood.

Mammals have three ADARs: *ADAR* (ADAR1), *ADARB1* (ADAR2), and *ADARB2* (ADAR3); although only ADAR1 and ADAR2 have editing activity in vitro. Physiologically, ADAR2 is essential for the specific editing of a single adenosine in the coding sequence of the glutamate receptor 2 subunit (*Gria2*) transcript, termed the Q/R site, encoding a neurotransmitter receptor in the brain [7–9]. Inosine is interpreted as guanine by the ribosome so editing of the *Gria2* mRNA recodes a genomically encoded glutamine (Q) to arginine (R), varying the permeability of the pore. *Adarb1* ^*-/-*^ (*Adar2* ^*-/-*^) mice cannot edit the Q/R site adenosine, leading to seizures and early lethality. Despite thousands of other editing events identified in both coding and non-coding regions, genomic substitution of the single A to G at the Q/R site rescued the *Adar2* ^*-/-*^ phenotype [7, 10]. This result defined the paradigm that RNA editing is a means to post-transcriptionally modify coding sequences and “recode” DNA sequences. Unlike ADAR2, the role of ADAR1 has proven more complex.

ADAR1 is expressed throughout the body, unlike ADAR2 which is highest in the brain [11]. There are two isoforms of ADAR1: a constitutively expressed ADAR1 p110, which is primarily nuclear-restricted, and a longer interferon (IFN) inducible ADAR1 p150, which localizes to both nucleus and cytoplasm [12]. *Adar* ^*-/-*^ (*Adar1* ^*-/-*^ *)* mice died at embryonic day 11.5–12.0 (E11.5–12.0) with a profound upregulation of type I IFN and IFN-stimulated genes (ISGs) [13–15]. Likewise, adult somatic deletion of *Adar1* resulted in innate immune activation and cell death [14, 16–19]. Mice homozygous for a single amino acid substitution resulting in an editing-deficient ADAR1 protein (*Adar1* ^*E861A/E861A*^) die at E13.5, with remarkably similar phenotypes to the null allele [19]. Activation of the innate immune sensing system, termed an interferonopathy, is observed in the subset of Aicardi-Goutières syndrome (AGS) patients who harbor mutations in *ADAR*, demonstrating that the transcriptional response to loss of ADAR1 activity is conserved across mammals [17, 20–22].

In mice and humans, most editing occurs in repetitive sequences capable of base-pairing to form intramolecular dsRNA structures [23–25]. Using murine genetics, we and others demonstrated that editing of endogenous RNA is a critical function of ADAR1, acting to prevent sensing of endogenous (self) dsRNA as foreign [17, 19, 26, 27]. A central function of the innate immune system is the recognition of foreign (non-self) dsRNA by pattern recognition receptors (PRRs) in the cytoplasm. Upon detecting long dsRNA, the PRR melanoma differentiation-associated gene 5 (MDA5, gene name *Ifih1*) oligomerizes to form a filament leading to activation of mitochondrial antiviral signaling (MAVS), resulting in the production of type I IFN and the propagation of downstream defence signals [28, 29]. Concurrent deletion of MDA5 or MAVS with ADAR1 extended the survival of *Adar1* ^*-/-*^ animals until shortly after birth [17, 26]. Viability of the ADAR1 editing-deficient mice was also rescued by deletion of MDA5. These findings suggested that RNA editing is the key function of ADAR1 during early development to prevent activation of MDA5 by endogenous dsRNA.

Editing-dependent and editing-independent functions of ADAR1 have been proposed, including involvement in multiple aspects of microRNA (miRNA) biogenesis [30–35], messenger RNA (mRNA) stability [36, 37], alternate 3’-UTR usage [33], splicing [38, 39] and translation [40]. Analysis of the *Adar1* ^*-/-*^ *Mavs* ^*-/-*^ and *Adar1p150* ^*-/-*^ *Mavs* ^*-/-*^ rescued mice identified phenotypes associated with the loss of ADAR1 that were considered independent of MAVS/MDA5, including developmental defects in the kidney, small intestine, lymph node, and a failure of B lymphopoiesis [17]. It is unclear if these phenotypes reflect editing-dependent or editing-independent functions of ADAR1.

Due to embryonic lethality of the *Adar1* ^*-/-*^ and *Adar1* ^*E861A/E861A*^ mice, establishing the in vivo relevance of ADAR1’s functions to adult homeostasis had not been possible. In contrast to the *Adar1* ^*-/-*^ *Ifih1* ^*-/-*^ or *Adar1* ^*-/-*^ *Mavs* ^*-/-*^ that die within days of birth, the *Adar1* ^*E861A/E861A*^ *Ifih1* ^*-/-*^ survived until at least four weeks of age [19]. We have assessed the requirement for ADAR1 activity during adult murine homeostasis using these rescued ADAR1 editing-deficient animals. We directly compared the *Adar1* ^*-/-*^ and *Adar1* ^*E861A*^ alleles, on both a *Ifih1* ^*+/+*^ and *Ifih1* ^*-/-*^ background, on the day of birth and in an acute adult somatic mutation model to evaluate editing-independent functions of ADAR1. We conclude that the core function of ADAR1-mediated editing is to prevent the formation of MDA5 substrates and that other effects of ADAR1-mediated editing, such as protein recoding and on miRNA biology, are not essential for murine homeostasis in vivo.

## Results

### Adar1^E861A/E861A^Ifih1^-/-^ animals have a normal lifespan and low weaning weight

**Fig. 1:**
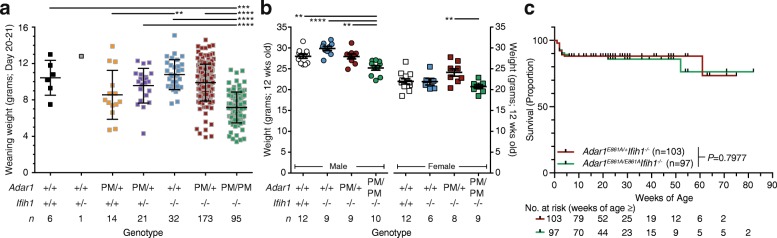
Reduced weaning weight and normal survival of *Adar1* ^*E861A/E861A*^ *Ifih1* ^*-/-*^ animals. **a** Weaning weight of indicated genotypes. **b** Weight of 12-week-old male and female animals of indicated genotypes. **c** Kaplan–Meier survival plot of *Adar1* ^*E861A/+*^ *Ifih1* ^*-/-*^ and *Adar1* ^*E861A/E861A*^ *Ifih1* ^*-/-*^. Number of animals at risk indicates the number in the cohort/age bracket. Results are mean ± SEM with *P < 0.05, **P < 0.005, and ****P < 0.00001

The *Adar1* ^*E861A/E861A*^ *Ifih1* ^*-/-*^ are viable [19]. The most apparent phenotype of the *Adar1* ^*E861A/E861A*^ *Ifih1* ^*-/-*^ animals was reduced weaning weight, with *Adar1* ^*E861A/E861A*^ *Ifih1* ^*-/-*^ animals ~ 30% lighter than the controls (day 20–21 post birth; Fig. 1a). By 12 weeks, the *Adar1* ^*E861A/E861A*^ *Ifih1* ^*-/-*^ females had a normal body weight compared to both wild-type (WT) C57BL/6 and *Adar1* ^*+/+*^ *Ifih1* ^*-/-*^ animals (Fig. 1b, Additional files 1 and 2: Video S1, S2). Males remained lighter at 12 weeks, although the difference was less than at weaning, being ~ 9% and ~ 17% lighter than C57BL/6 or *Adar1* ^*+/+*^ *Ifih1* ^*-/-*^, respectively (Fig. 1c, Additional files 1 and 2: Video S1, S2). *Adar1* ^*E861A/+*^ *Ifih1* ^*-/-*^ animals had normal body weight. There was no significant difference in the long-term survival of the *Adar1* ^*E861A/E861A*^ *Ifih1* ^*-/-*^ animals compared to *Adar1* ^*E861A/+*^ *Ifih1* ^*-/-*^ animals, surviving to > 80 weeks of age (Fig. 1c). The oldest *Adar1* ^*E861A/E861A*^ *Ifih1* ^*-/-*^ animals have survived to > 115 weeks of age (>805 days). The median life expectancy of C57BL/6 mice is in the range of 676–878 days [41, 42]. Both male and female *Adar1* ^*E861A/E861A*^ *Ifih1* ^*-/-*^ animals are fertile, which for the males, at least, this is consistent with the primary role reported for ADAR2 in editing in the testis [43].

### Mild innate immune activation in the absence of MDA5

**Fig. 2:**
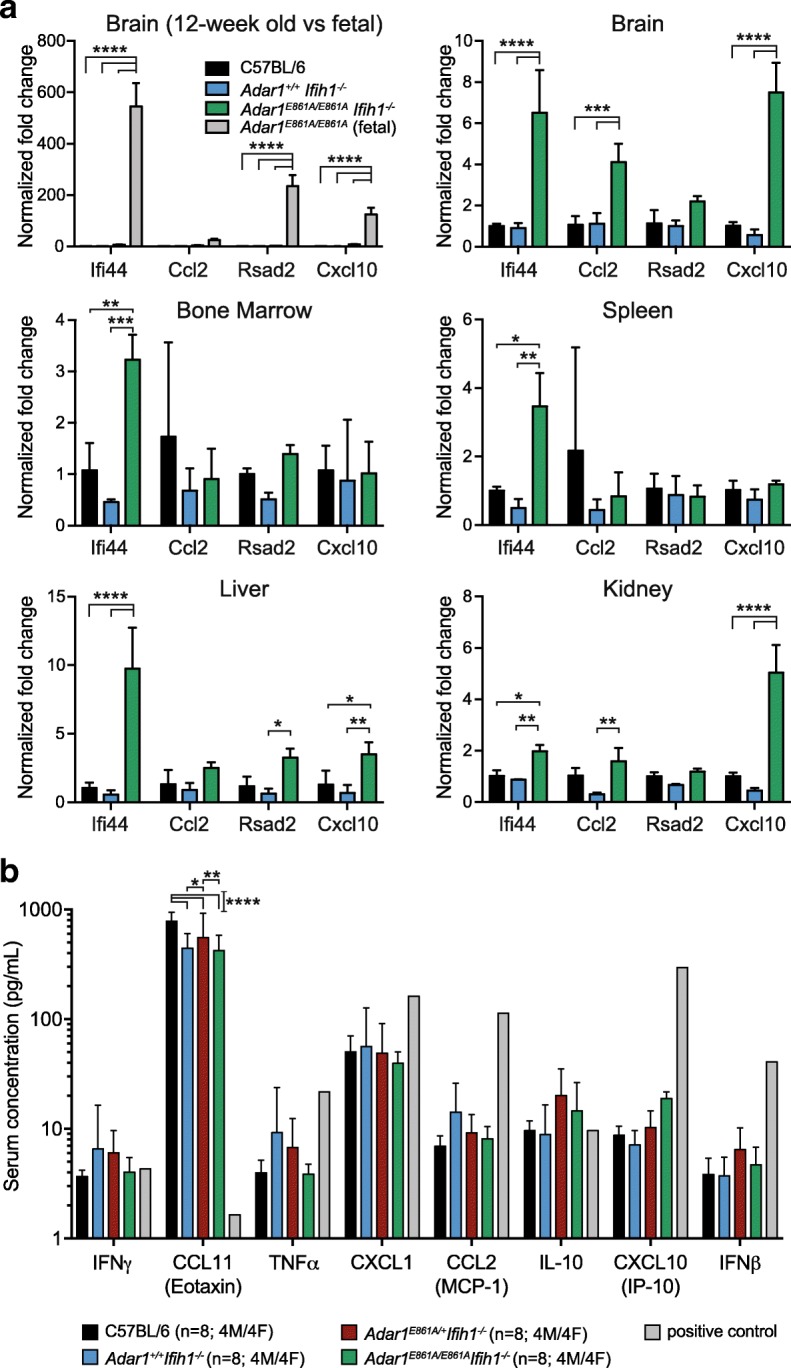
Modest activation of ISG expression in *Adar1* ^*E861A/E861A*^ *Ifih1* ^*-/-*^ tissues. **a** Quantitative reverse transcription polymerase chain reaction (qRT-PCR) of four ISGs from tissues of 12-week-old mice, with the exception of the first panel comparing 12-week-old brain with E12.5 fetal brain from *Adar1* ^*E861A/E861A*^ *Ifih1* ^*+/+*^ embryos. Data represent mean ± SEM (n = 3/genotype); all samples normalized to C57BL/6. **b** PB serum cytokine levels from indicated genotypes. Positive control = supernatant from tamoxifen treated *R26*-CreER^T2^ *Adar1* ^*E861A/fl*^ hematopoietic cell line. Results are mean ± SEM; significance determined using two-way ANOVA, *P < 0.05, **P < 0.01, ***P < 0.001, ****P < 0.0001

To determine if there was activation of innate immune sensing in the *Adar1* ^*E861A/E861A*^ *Ifih1* ^*-/-*^ animals, as seen in the *Adar1* ^*-/-*^ [14] and the *Adar1* ^*E861A/E861A*^ [19], the expression of four ISGs was assessed across a range of tissues (Fig. 2a). The expression of these ISGs increased dramatically upon deletion/mutation of ADAR1 in both human and mouse [17, 21]. There was either no change or a subtle increase in ISGs in the tissues assessed (up to tenfold over C57BL/6 s). In whole brain, three of four ISGs were significantly increased. By comparison, in E12.5 *Adar1* ^*E861A/E861A*^ vs. *Adar1* ^*+/+*^ fetal brain, these same ISGs were upregulated between ~ 125 and ~ 550-fold (Fig. 2a). Therefore, MDA5 is the primary conduit of innate immune signal activation in ADAR1 editing-deficient mice. However, a well-tolerated and, apparently non-pathogenic, low-level, innate immune activation occurs in the absence of MDA5 via currently unknown mechanisms.

The protein levels of eight cytokines/chemokines in peripheral blood (PB) serum that increased upon somatic deletion of *Adar1* were assessed [17]. There was no increase in any of the proteins assessed including IFNβ, IFNγ, CCL2, and CXCL10 (Fig. 2b). The only difference was an *Ifih1* ^*-/-*^ genotype-dependent reduction in the expression of CCL11 (Fig. 2b). As a positive control, the expression of these proteins was assessed in supernatant from cultures of immortalized hematopoietic cells [44], generated from *R26*-CreER^T2^ *Adar1* ^*E861A/fl*^ bone marrow (BM) cells, treated with tamoxifen in vitro (Fig. 2b). Despite the increased ISG mRNAs there is no resultant increase in circulating protein levels.

### Normal hematopoiesis in the absence of ADAR1-mediated A-to-I editing

**Fig. 3:**
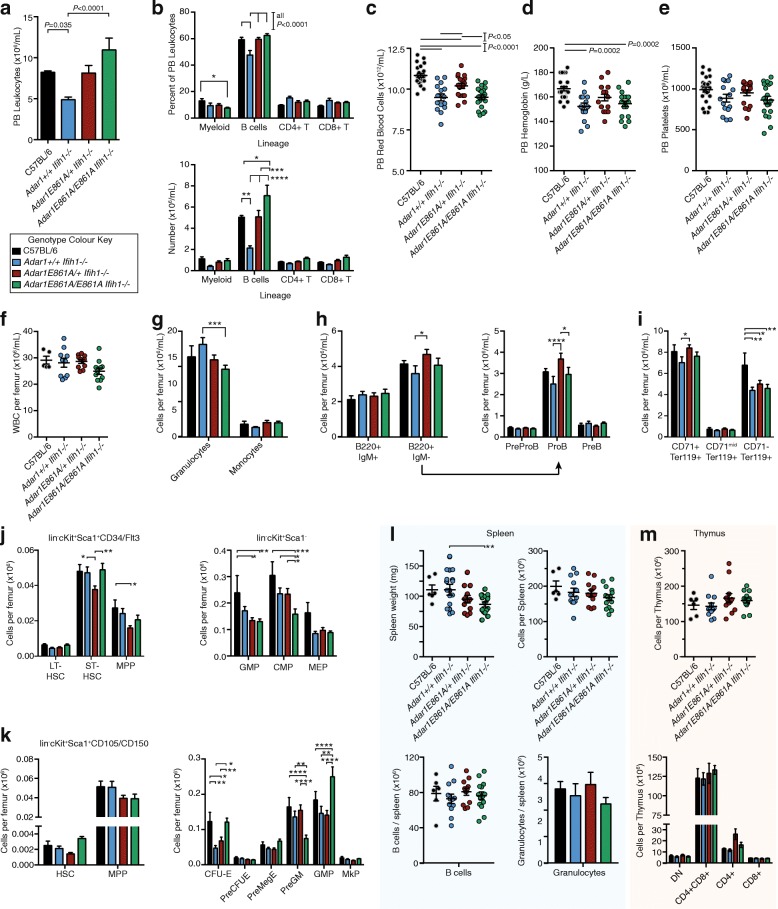
Hematopoiesis and B-cell production are normal in the absence of ADAR1 editing. PB, BM, spleen, and thymus analyzed of 12-week-old mice. **a**–**e** PB analysis from C57BL/6 (n = 25), *Adar1* ^*+/+*^ *Ifih1* ^*-/-*^ (n = 15), *Adar1* ^*E861A/+*^ *Ifih1* ^*-/-*^ (n = 17), *Adar1* ^*E861A/E861A*^ *Ifih1* ^*-/-*^ (n = 19) indicating (**a**) total leukocyte counts, (**b**) % and absolute number of each leukocyte subtype, (**c**) red blood cell counts, (**d**) hemoglobin levels, and (**e**) platelet numbers. **f**–**j** BM analysis of C57BL/6 (n = 5), *Adar1* ^*+/+*^ *Ifih1* ^*-/-*^ (n = 10), *Adar1* ^*E861A/+*^ *Ifih1* ^*-/-*^ (n = 11), *Adar1* ^*E861A/E861A*^ *Ifih1* ^*-/-*^ (n = 11) showing (**f**) total leukocyte counts per femur, (**g**) numbers of granulocytes and monocytes, (**h**) mature (B220 + IgM+) and immature (B220 + IgM-) B-cell populations and subsets of the immature populations as indicated, (**i**) erythroid cells; and **j**–**k** hematopoietic stem and progenitor populations using two methods. (**l**) Spleen weight and cellularity, B cells and granulocyte numbers per spleen from C57BL/6 (n = 6), *Adar1* ^*+/+*^ *Ifih1* ^*-/-*^ (n = 12), *Adar1* ^*E861A/+*^ *Ifih1* ^*-/-*^ (n = 12), *Adar1* ^*E861A/E861A*^ *Ifih1* ^*-/-*^ (n = 14). (**m**) Thymic cellularity and CD4/CD8 composition in C57BL/6 (n = 6), *Adar1* ^*+/+*^ *Ifih1* ^*-/-*^ (n = 12), *Adar1* ^*E861A/+*^ *Ifih1* ^*-/-*^ (n = 12), *Adar1* ^*E861A/E861A*^ *Ifih1* ^*-/-*^ (n = 13). Results are mean ± SEM; data shown are pooled from at least three independent experiments; significance determined using two-way ANOVA with correction for multiple comparisons; *P < 0.05, **P < 0.01, ***P < 0.001, ****P < 0.0001

Hematopoietic cells have a profound dependence on ADAR1 [13, 14, 16, 18, 19, 45]. We assessed hematopoiesis in 12-week-old *Adar1* ^*E861A/E861A*^ *Ifih1* ^*-/-*^ animals and three control populations: age-/sex-matched C57BL/6 animals bred and housed in the same facility; *Adar1* ^*+/+*^ *Ifih1* ^*-/-*^ animals; and *Adar1* ^*E861A/+*^ *Ifih1* ^*-/-*^ animals. PB indices in *Adar1* ^*E861A/E861A*^ *Ifih1* ^*-/-*^ animals were comparable to C57BL/6 mice (Fig. 3a). For reasons presently unknown, the total PB leukocyte counts were lower in the *Adar1* ^*+/+*^ *Ifih1* ^*-/-*^ cohort compared to the other genotypes (Fig. 3a, b). There were several statistically significant differences between the *Adar1* ^*E861A/E861A*^ *Ifih1* ^*-/-*^ animals and C57BL/6 animals including a reduction in the percentage, but not numbers, of granulocytes and an increase in the number, but not percentage, of B cells in the PB (Fig. 3b). All *Ifih1* ^*-/-*^ animals, irrespective of *Adar1* status, presented with a mild anemia compared to the C57BL/6 cohorts (Fig. 3c, d). Platelet numbers were normal in all genotypes (Fig. 3e).

Within the BM, relatively mild and subtle changes were apparent that reflected the PB. Total BM cellularity and myeloid development was essentially normal between genotypes (Fig. 3f, g). Analysis of B-cell differentiation demonstrated that the lower B-cell numbers in the *Adar1* ^*+/+*^ *Ifih1* ^*-/-*^ stemmed from a reduction of cells at the ProB cell stage (Fig. 3h). Consistent with the *Ifih1* ^*-/-*^-dependent anemia, there were reduced numbers of mature CD71^-^Ter119^+^ erythroid cells in the BM of animals with this genotype (Fig. 3i). In the stem and primitive progenitor populations, there were relatively subtle differences among the groups, but in all cases the numbers of each population were largely normal (Fig. 3j, k). Hematopoiesis in the spleen (Fig. 3l) and thymus (Fig. 3m) was normal compared to controls. ADAR1-mediated editing in not required for homeostatic hematopoiesis once MDA5 is inactivated.

### Normal tissue development in the absence of ADAR1-mediated A-to-I editing

**Fig. 4:**
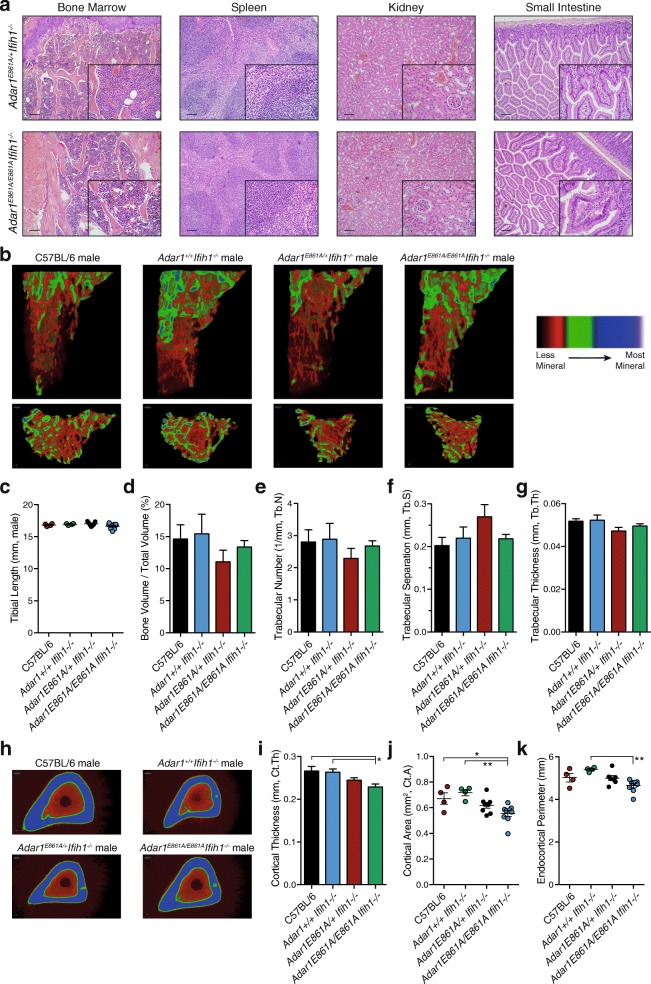
Normal organ histology in the absence of ADAR1 RNA editing. **a** Hematoxylin and eosin (H&E)-stained sections of the indicated organs of *Adar1* ^*E861A/E861A*^ *Ifih1* ^*-/-*^ (n = 4; 2 male, 2 female) and *Adar1* ^*E861A/+*^ *Ifih1* ^*-/-*^ (n = 4; 2 male, 2 female) control littermates. 10× magnification with 100-μM scale bar; 40× inset for each. **b**–**g** MicroCT analysis of tibial bone; C57BL/6 (n = 4), *Adar1* ^*+/+*^ *Ifih1* ^*-/-*^ (n = 4), *Adar1* ^*E861A/+*^ *Ifih1* ^*-/-*^ (n = 7), *Adar1* ^*E861A/E861A*^ *Ifih1* ^*-/-*^ (n = 9). **b** Representative images of reconstructed trabecular region of the secondary spongiosa within the proximal tibia with color-coded quantitative mineralization from indicated genotypes. **c** Tibial length, (**d**) Trabecular bone volume, (**e**) trabecular number, (**f**) trabecular separation, and (**g**) trabecular thickness. **h**–**k** MicroCT analysis of cortical bone of the same samples used for panels (**b**–**g**). **h** Representative images of reconstructed cortical bone with color-coded quantitative mineralization, (**i**) cortical thickness, (**j**) cortical bone area, and (**k**) endocortical perimeter. Results are mean ± SEM; significance tested using ANOVA, *P < 0.05, **P < 0.01

Tissues from 12-week-old *Adar1* ^*E861A/E861A*^ *Ifih1* ^*-/-*^ and age-/sex-matched *Adar1* ^*E861A/+*^ *Ifih1* ^*-/-*^ littermates were subjected to a genotype blinded histological assessment by an independent core service facility. No significant pathology or differences were observed across the > 20 tissues and structures assessed between *Adar1* ^*E861A/E861A*^ *Ifih1* ^*-/-*^ to *Adar1* ^*E861A/+*^ *Ifih1* ^*-/-*^ littermates. The kidney, spleen, and small intestine were normal (Fig. 4a). All other organs assessed, including brain, were histologically normal (see Additional file 3: Dataset S1).

Due to the reduced weaning weight and requirement for ADAR1 in bone/osteoblast homeostasis [46], we quantitated the bone parameters in 12-week-old *Adar1* ^*E861A/E861A*^ *Ifih1* ^*-/-*^ animals compared to controls by microCT [47]. Both male and female mice were assessed; however, due to variability in bone indices with estrus in females, we focused our analysis on the males (female data in Additional file 3: Figure S1). The total tibial length and trabecular bone parameters were normal in the *Adar1* ^*E861A/E861A*^ *Ifih1* ^*-/-*^ animals (Fig. 4b–g). The only significant differences were modest changes in the cortical thickness (Fig. 4h, i) and cortical area (Fig. 4j) between the *Adar1* ^*E861A/E861A*^ *Ifih1* ^*-/-*^ animals and the C57BL/6 and *Adar1* ^*+/+*^ *Ifih1* ^*-/-*^ animals. There was a reduced endocortical perimeter when the *Adar1* ^*E861A/E861A*^ *Ifih1* ^*-/-*^ were compared to the *Adar1* ^*+/+*^ *Ifih1* ^*-/-*^ animals (Fig. 4k), but not to the C57BL/6 s. Therefore, ADAR1-mediated A-to-I editing is dispensable for normal organ and skeletal development once MDA5 is inactivated.

### Transcriptome analysis of whole brain from adult rescued mice

**Fig. 5:**
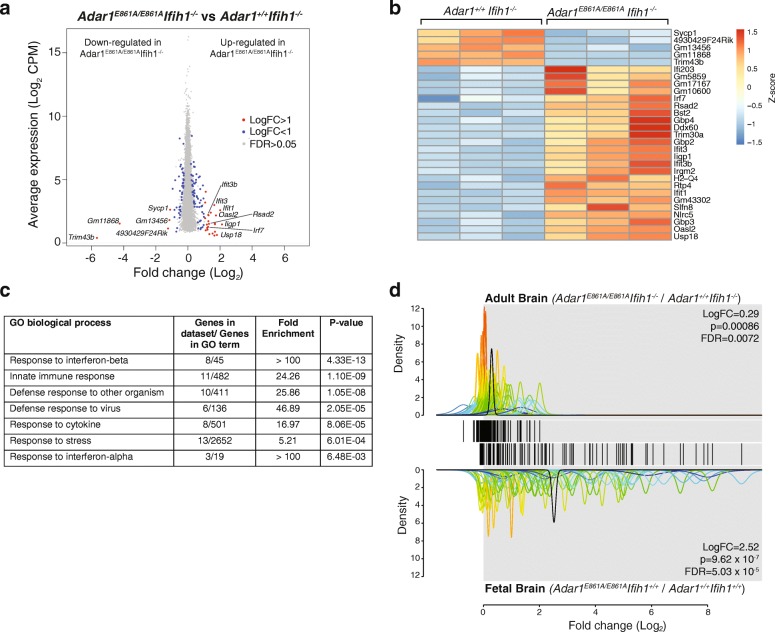
The absence of ADAR1-mediated editing has minimal effect on gene expression in the adult brain. RNA-sequencing (RNA-seq) analysis of 12-week-old mouse brains (n = 3 biological replicates/genotype). **a** MA plot for differentially expressed genes. All genes expressed in brain with CPM ≥ 2 in all replicates/genotype indicated by a *dot*. **b** *Heatmap* of differentially expressed genes with a log_2_FC ≥ 1 in three biological replicates/genotype. *Colors* reflect the z-score. **c** Enriched pathways by GO term within differentially expressed genes (false discovery rate [FDR] ≤ 0.05, log_2_FC ≥ 1, n = 29 genes). **d** QuSAGE analysis using the *Adar1* ^*E861A/E861A*^ fetal liver gene signature as the gene set. Each gene is depicted by a single line within the barcode. Adult brain from *Adar1* ^*E861A/E861A*^ *Ifih1* ^*-/-*^ mice and fetal brain from *Adar1* ^*E861A/E861A*^ mice (relative to respective controls; n = 3 biological replicates/genotype) were compared against the *Adar1* ^*E861A/E861A*^ gene set. *Curves* represent the standard deviation between biological replicates for each gene and are *color-coded* by the magnitude of deviation. The *black curve* in each panel represents the average log_2_FC for all genes in the set in each dataset

*ADAR1* loss-of-function mutations in humans are one of the causes of AGS [17, 20, 21]. AGS is characterized by severe changes in the brain and neurological function, so we sought to understand how a loss of editing by ADAR1 impacted the brain transcriptome. Whole brains of 12-week-old *Adar1* ^*E861A/E861A*^ *Ifih1* ^*-/-*^ and *Adar1* ^*+/+*^ *Ifih1* ^*-/-*^ male mice were assessed (Fig. 5a, Additional file 4: Dataset S2). Compared to that previously observed in the *Adar1* ^*-/-*^ and *Adar1* ^*E861A/E861A*^ fetal tissues where the expression of many hundreds of transcripts is altered [14, 19], there were surprisingly limited changes in the transcriptome of the *Adar1* ^*E861A/E861A*^ *Ifih1* ^*-/-*^ brain. Twenty-nine genes were significantly different between the genotypes (log_2_FC ≥ 1, ≥ 2 counts per million [CPM] in all three replicates/genotype). Most changes (n = 24) were transcripts upregulated in the absence of editing (Fig. 5b). Pathway analysis confirmed the modest innate immune activation signature, as observed with quantitative polymerase chain reaction (qPCR), with GO pathways relating to IFN response and viral defence the only pathways enriched in the differentially expressed genes (Fig. 5c; Additional file 4: Dataset S2). Quantitative set analysis for gene expression (QuSAGE) was performed to examine the transcriptional signature of ADAR1-editing deficiency, defined in our previous analysis of the *Adar1* ^*E861A/E861A*^ fetal liver [19], in the adult brains (Fig. 5d). To do this, differentially expressed genes in the E12.5 *Adar1* ^*E861A/E861A*^ fetal liver were used to create a gene set and the expression of the genes in this set were assessed in both the adult brain of *Adar1* ^*E861A/E861A*^ *Ifih1* ^*-/-*^ (vs. *Adar1* ^*+/+*^ *Ifih1* ^*-/-*^) and, as a comparator, the E12.5 fetal brain of *Adar1* ^*E861A/E861A*^ embryos (vs. *Adar1* ^*+/+*^; Fig. 5d, Additional file 4: Dataset S2). The ADAR1-editing deficient signature is enriched in the adult brain even in the absence of MDA5, however, at a greatly reduced level compared to the MDA5 WT *Adar1* ^*E861A/E861A*^ E12.5 fetal brain.

### ADAR1 is the primary editing enzyme in peripheral tissue and editing levels are significantly reduced in the Adar1^E861A/E861A^Ifih1^-/-^ tissues

**Fig. 6:**
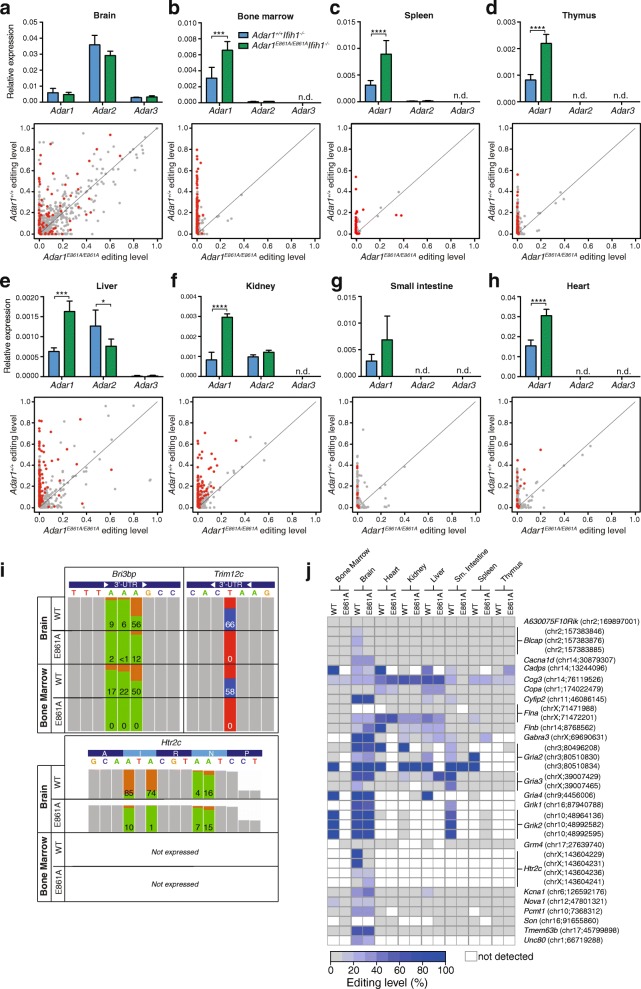
ADAR1 is the predominant ADAR in peripheral tissues and affects editing levels across all tissues. **a**–**h** *Upper panels*: qRT-PCR of *Adar* expression from tissues of 12-week-old mice. Data normalized to *Ppia* and are the mean ± SEM (n = 3/genotype). *n.d* not detected. *Lower panels*: Editing of known sites measured using multiplexed PCR and deep sequencing (mmPCR-seq) in each tissue. Editing levels in *Adar1* ^*+/+*^ *Ifih1* ^*-/-*^ (ADAR1 WT; *y-axis*) plotted against those in the *Adar1* ^*E861A/E861A*^ *Ifih1* ^*-/-*^ (ADAR1 editing deficient; *x-axis*) with each individual site indicated by a *dot. Gray dot* = no significant difference, *red dot* = *P* < 0.05. **i** Examples of editing at three loci. *Colored panels* indicate the edited nucleotides ((+) strand: *green* = A, *orange* = G; (-) strand: *red* = T(A), *blue* = C(G)). *Bri3bp* is a representative shared *Adar1* and *Adar2* target at the third adenosine; *Trim12c* is representative of an ADAR1-specific target. *Htr2c* is representative of an ADAR1-specific recoding event at the first two sites. Percent editing is indicated for each site. **j** Analysis of conserved editing sites in each tissue by mmPCR-seq. Editing sites were compared between *Adar1* ^*+/+*^ *Ifih1* ^*-/-*^ (ADAR1 WT) and *Adar1* ^*E861A/E861A*^ *Ifih1* ^*-/-*^ tissues as indicated. *White* indicates no expression/reads detected in the tissue. *Data* include all sites with > 100 reads in the mmPCR-seq

One potential explanation for the normality of the rescued animals could be redundancy for editing targets with ADAR2. We therefore assessed eight tissues for the expression of *Adar* isoforms and editing of known sites: BM, spleen, thymus, liver, brain, kidney, small intestine, and heart. *Adar1* transcript could be detected in all tissues, whereas *Adar2* transcript was expressed only in brain, kidney, and liver with low levels in spleen and BM (Fig. 6a–h, upper panels). *Adar3* transcript was detected in brain and at low levels in the liver only. Modestly increased overall expression of *Adar1* was observed in *Adar1* ^*E861A/E861A*^ *Ifih1* ^*-/-*^ relative to *Adar1* ^*+/+*^ *Ifih1* ^*-/-*^ in BM, spleen, thymus, liver, kidney, and heart, possibly reflecting the mild ISG activation observed (Fig. 4a).

A-to-I editing across a range of defined substrates was assessed using microfluidics multiplexed PCR and deep sequencing (mmPCRseq) [48], allowing accurate measurements of editing of up to 11,103 sites in 557 loci (Fig. 6a–h, lower panels, Additional file 5: Dataset S3). Changes in editing across the tissues principally reflected the expression profile of *Adar1* and *Adar2*. In the brain, where there are higher relative levels of *Adar2* than *Adar1*, there is a largely preserved editome with evidence for a subset of ADAR1-specific and ADAR2-specific events (dots on y-axis or x-axis, respectively), but the majority can be edited by both. In contrast, in hematopoietic tissues there is little/no detectable *Adar2* and with this there is a near complete absence of editing of known sites when ADAR1 editing is inactivated. In other peripheral tissues assessed, *Adar1* predominates and most editing of known sites was lost in the *Adar1* ^*E861A/E861A*^-derived samples. Examples for ADAR1-specific, ADAR2-specific, and ADAR1/2 shared editing sites were observed (Fig. 6i). We further assessed the status of evolutionary conserved recoding events across the tissues [49]. The most informative tissue was the brain, where the majority of these transcripts were robustly expressed (Fig. 6j). In the brain, editing at most sites was preserved indicating that ADAR2 was editing these sites. Known ADAR1 dependent sites such as *Bclap* and two sites in the *Htr2c* transcript had significantly reduced/no editing in the brain [13, 50]. For the remaining tissues, there were several patterns apparent: the transcripts were not detected as expressed in the tissue assessed (indicated in white; see Additional file 5: Dataset S3 for mouse ENCODE expression data from tissues for each transcript); that editing of the conserved sites was preserved indicating an ADAR2-specific target, even in tissue with relatively low levels of *Adar2* as assessed by qPCR (see *Cog3* as an example); that there was a loss of editing at these sites in the *Adar1* ^*E861A/E861A*^ *Ifih1* ^*-/-*^-derived tissues indicating these sites are ADAR1-dependent (see *Kcna1* in the liver, *Nova1* in the bone marrow, *Cyfip2* in the liver and small intestine for examples). The failure to edit the conserved sites in the *Adar1* ^*E861A/E861A*^ *Ifih1* ^*-/-*^ tissues suggests that while they are ADAR1-dependent sites, their absence was not deleterious. This analysis demonstrated that editing in the tissues outside the central nervous system that were assessed is primarily mediated by ADAR1, yet despite the loss of this editing, these tissues are normal in the absence of MDA5.

### The absence of ADAR1 or loss of ADAR1-mediated A-to-I editing are equivalent in vivo

**Fig. 7:**
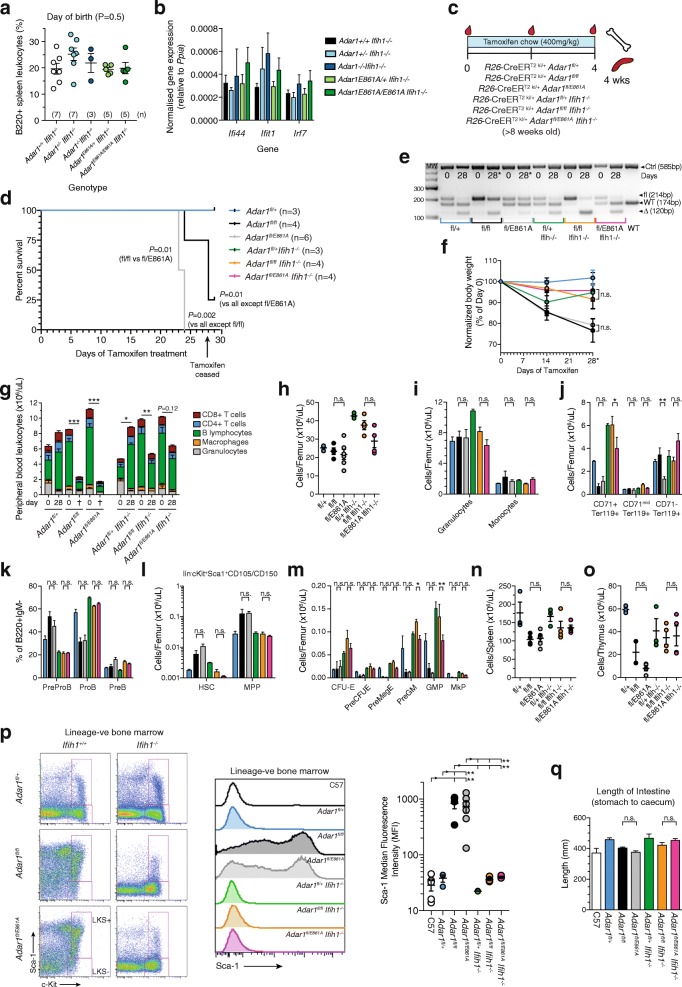
The complete absence of ADAR1 and the specific loss of ADAR1-mediated editing activity phenocopy. **a** Percentage of B cells (B220 + ve) spleen leukocytes in the indicated genotypes at the day of birth, n as indicated in (**a**). Data are pooled from at least two separate litters per genotype. **b** Expression of *Ifi44*, *Ifit1*, and *Irf7* transcript in whole brain from the indicated genotypes on the day of birth, n as for (**a**). **c** Experimental outline of somatic deletion model; all mice were aged ≥ 8 weeks at tamoxifen initiation (defined as day 0). **d** Kaplan–Meier survival *plot* of each genotype. All *Ifih1* ^*-/-*^ lines fall under the *Adar1* ^*fl/+*^. Mice were analyzed on the day of euthanasia or day 29 as indicated. **e** Genotyping of genomic DNA at day 0 (PB-derived cells) and day 28/euthanasia (BM-derived cells) for each genotype. **f** Change in body weight (day 0 normalized to 100%) and day 28/euthanasia. **g** PB leukocyte counts and lineage distribution within the total leukocyte count at day 0 (pre-tamoxifen) and day of euthanasia or day 28 as indicated in (**b**). Statistics compare day 0 and day of euthanasia/day 28 within an individual genotype. **h** BM cellularity, (**i**) granulocytes and macrophages, (**j**) erythroid cells, (**k**) percentages of B cell precursors within the B220 + IgM- population, (**l**) stem cell and multipotent progenitor populations, (**m**) the numbers of myelo-erythroid progenitors/femur for each genotype. **n** Spleen and (**o**) thymus cellularity. **p** Representative FACS *plots* of Sca-1 expression in the lin^-^c-Kit^+^ BM fraction. Representative median fluorescence intensity histogram *plots* of Sca1 and quantitation of Sca1 expression levels. **q** Intestine length (stomach – caecum) for each genotype at day of euthanasia or day 29 as indicated in (**b**). Statistical comparison (except (**b**, **d**)) shown only for *R26*-CreER^ki/+^ *Adar1* ^*fl/fl*^ vs. *R26*-CreER^ki/+^ *Adar1* ^*fl/E861A*^ and *R26*-CreER^ki/+^ *Adar1* ^*fl/fl*^ *Ifih1* ^*-/-*^ vs. *R26*-CreER^ki/+^ *Adar1* ^*fl/E861A*^ *Ifih1* ^*-/-*^. Full statistical analysis of all comparisons in Additional file 6: Table S1. Number of animals per group indicated in (**b**); data are pooled from two separate experiments. *P < 0.05; **P < 0.01, ***P < 0.001

A direct comparison of the specific loss of ADAR1-mediated editing and complete absence of ADAR1 protein would clarify the potential for ADAR1 to contribute to processes beyond A-to-I editing. The difference in viability and tissue phenotypes between *Adar1* ^*-/-*^ *Ifih1* ^*-/-*^ and *Adar1* ^*E861A/E861A*^ *Ifih1* ^*-/-*^ rescued mice suggested there may be editing-independent roles of ADAR1. We initially assessed cohorts of *Adar1* ^*-/-*^ *Ifih1* ^*-/-*^ and *Adar1* ^*E861A/E861A*^ *Ifih1* ^*-/-*^ pups and littermate controls at the day of birth, a time point when the majority of the *Adar1* ^*-/-*^ *Ifih1* ^*-/-*^ are viable [17]. At this developmental age, there was no difference between the ADAR1 null and editing-deficient alleles with the frequency of B cells in the spleen being equivalent and no apparent activation of the innate immune sensing pathway (Fig. 7a, b).

Next, we evaluated this using an acute somatic deletion model comparing *R26*-CreER *Adar1* ^*fl/+*^ (control; expressing WT ADAR1 protein after tamoxifen treatment), *R26*-CreER *Adar1* ^*fl/fl*^ (ADAR1 protein null after tamoxifen treatment), and *R26*-CreER *Adar1* ^*fl/E861A*^ (expressing an editing deficient ADAR1 protein after tamoxifen treatment) on both an MDA5 WT and null background (Fig. 7c). All mice were aged at least eight weeks at the initiation of up to 28 days treatment with tamoxifen administered via the food (400 mg/kg in standard chow). The *R26*-CreER *Adar1* ^*fl/fl*^ and *R26*-CreER *Adar1* ^*fl/E861A*^ animals both lost condition and weight from day 14 onward (Fig. 7d–f). This decline in health was accompanied by a severe reduction in PB indices and the euthanasia of all *R26*-CreER *Adar1* ^*fl/E861A*^ animals and three of four *R26*-CreER *Adar1* ^*fl/fl*^ animals prior to day 28 of treatment (Fig. 7f, g). All remaining genotypes remained healthy at day 28 and were assessed on day 29 (Fig. 7d). Upon euthanasia or cessation of tamoxifen as indicated, genotyping confirmed the efficient recombination of the floxed allele in the *R26*-CreER *Adar1* ^*fl/+*^ animals (Fig. 7e). The *R26*-CreER *Adar1* ^*fl/fl*^ and *R26*-CreER *Adar1* ^*fl/E861A*^, while both moribund and requiring euthanasia, retained the floxed allele indicative of selection against deletion as previously observed in other settings [14, 18, 19]. Strikingly, the absence of MDA5 allowed the efficient recombination of the floxed allele in both the *R26*-CreER *Adar1* ^*fl/fl*^ *Ifih1* ^*-/-*^ and *R26*-CreER *Adar1* ^*fl/E861A*^ *Ifih1* ^*-/-*^ animals (Fig. 7e).

We focused our assessment on hematopoiesis, given the well-characterized impact of ADAR1 deficiency on this system. Across PB (Fig. 7g), BM (Fig. 7h–m), spleen (Fig. 7n, Additional file 3: Figure S2), and thymus (Fig. 7o, Additional file 3: Figure S2), the results were highly comparable: that the complete absence of ADAR1 protein and the specific absence of A-to-I editing by ADAR1 were largely indistinguishable, and that the deletion of MDA5 rescued both the ADAR1 null and editing deficient alleles equivalently. In the presence of MDA5, the deletion of ADAR1 or prevention of editing by ADAR1 resulted in a loss of erythroid progenitors (Fig. 7j), a block in B-cell maturation in the BM (Fig. 7k), an accumulation of phenotypic stem and primitive progenitor populations (Fig. 7l) with a loss of the committed myelo-erythroid progenitor populations (Fig. 7m). These changes were prevented by the deletion of MDA5. The activation status of the innate immune response was queried using expression of Sca1, a cell surface protein induced by IFN resultant from an innate immune response [14, 51]. In the *R26*-CreER *Adar1* ^*fl/fl*^ and *R26*-CreER *Adar1* ^*fl/E861A*^ BM primitive hematopoietic population, there was a profound upregulation of Sca1 expression that was completely prevented by deletion of MDA5 (Fig. 7p). Intestinal length was assessed as prior work identified intestinal shortening following the somatic deletion of *Adar1* in a *Mavs* ^*-/-*^ background, although the age of these animals was not provided [17]. No difference in intestinal length was observed in our cohorts (Fig. 7q). These results demonstrate that the complete absence of ADAR1 and the specific absence of ADAR1-mediated A-to-I editing, in either the presence or absence of MDA5, respectively, are largely indistinguishable in adult mice. Therefore, preventing MDA5 substrate formation requires A-to-I editing of endogenous RNA and is the primary in vivo function of ADAR1.

## Discussion

The consequences of altered ADAR1 function are severe, from embryonic lethality in mice to debilitating neurological disease and systemic interferonopathy in humans with loss-of-function alleles [22, 52], to putative oncogenic roles when overexpressed [31, 53, 54], so it is critical to clearly define the key function(s) of ADAR1. In contrast to the physiologically essential role of transcript recoding by ADAR2, the importance of recoding to the biology of ADAR1 was unknown. In addition to protein recoding, ADAR1 can edit dsRNA substrates resulting in changes in multiple aspects of miRNA biogenesis or function, affect mRNA stability, 3’-UTR length and translation, and modify splice site usage in addition to altering dsRNA secondary structures, which have been proposed to interface with the innate immune sensing system [19, 55]. We now demonstrate that the absence of ADAR1-mediated editing is surprisingly well tolerated, once the innate immune sensor MDA5 is deleted. *Adar1* ^*E861A/E861A*^ *Ifih1* ^*-/-*^ mice are strikingly normal in a homeostatic state. Given the extent of A-to-I editing in the transcriptome, the precedent of ADAR2/*Gria2* and the diverse functions in which ADAR1 is implicated [2, 6, 56, 57], this was unexpected. This demonstrates that exonic recoding of transcripts by ADAR1, as well as other proposed editing-dependent effects of ADAR1 on gene expression, are not essential for development and adult homeostasis. Consistent with this, we saw very few changes to the global gene expression profile in adult brain of *Adar1* ^*E861A/E861A*^ *Ifih1* ^*-/-*^ mice. The lack of phenotypes in the *Adar1* ^*E861A/E861A*^ *Ifih1* ^*-/-*^ mice at homeostasis reveals that ADAR1’s primary and physiologically most important function is to edit endogenous RNA to prevent the formation of endogenous MDA5 substrates.

We do not exclude subtle or additional phenotypes in the *Adar1* ^*E861A/E861A*^ *Ifih1* ^*-/-*^ mice that were not appreciated in our analysis, as were demonstrated after the broad phenotypic testing of the *Adar2* ^*-/-*^ *Gria2* ^*R/R*^ mice [10]. Additionally, the analysis of the *Adar1* ^*E861A/E861A*^ *Ifih1* ^*-/-*^ animals in non-homeostatic settings, such as when subjected to stress or in pathological settings, may identify essential functions for ADAR1-mediated editing that are not apparent under the homeostatic conditions assessed here. A small number of mammalian conserved, positively selected recoding events have been identified [49]. While ADAR2 can edit many of these sites, particularly in the brain, it is also highly likely that the requirement for these may only become apparent in very specific circumstances. The known conserved ADAR1 dependent sites in *Bclap* and the *Htr2c* transcript were no longer edited in the *Adar1* ^*E861A/E861A*^ *Ifih1* ^*-/-*^ animals. Recoding events mediated by ADAR1 have been demonstrated to result in altered protein function. An example is the recoding of a serine-to-glycine in *AZIN1* (p.S365G) [49, 54]. In this instance, the edited form of AZIN1 is predicted to result in a gain-of-function allele, whereas our murine models have a loss of ADAR1-mediated editing and the physiological consequences of this are likely to be distinct. Overall, the absence of the conserved ADAR1-mediated recoding events appears well tolerated, suggesting that these proteins may have functions in the whole organism that are either mild/subtle, that their absence is not sufficient to be pathogenic in isolation, or that these only demonstrate an essential requirement in specific settings.

The nexus between ADAR1 and the innate immune system, centered on the MDA5-MAVS axis, has solidified due to the work of a number of groups, including our own [17, 26]. However, the differences between the *Adar1* ^*E861A/E861A*^ *Ifih1* ^*-/-*^ animals reported here and the various *Adar1* ^*-/-*^ rescued mice are intriguing [17]. Mannion et al. reported survival of the *Adar1* ^*-/-*^ *Mavs* ^*-/-*^ animals until the day of birth and that there was normal histology of internal organs of an *Adar1* ^*-/-*^ *Mavs* ^*-/-*^ pup at this time [26]. Pestal et al. also identified that the vast majority (>90–95%) of both *Adar1* ^*-/-*^ *Mavs* ^*-/-*^ and *Adar1* ^*-/-*^ *Ifih1* ^*-/-*^ pups died by two days after birth. A very small number of the *Adar1* ^*-/-*^ *Mavs* ^*-/-*^ pups were identified that survived for 13–20 days. These rare *Adar1* ^*-/-*^ *Mavs* ^*-/-*^ survivors had defects in kidney patterning, small intestines, lymph nodes, and B lymphopoiesis [17]. The *Adar1* ^*E861A/E861A*^ *Ifih1* ^*-/-*^ animals do not have these phenotypes, nor is an absence of B cells apparent in the cohort of *Adar1* ^*-/-*^ *Ifih1* ^*-/-*^ pups we assessed on the day of birth (Fig. 7a, b). The simplest interpretation of this is that editing-independent functions of ADAR1 have essential functions, specifically in early post-natal development. The phenotypes reported for the rescued *Adar1* ^*-/-*^ were on a *Mavs* ^*-/-*^ background, which may be an important contributing factor to the differences compared to the *Adar1* ^*E861A/E861A*^ *Ifih1* ^*-/-*^. The genetic rescue of the *Adar1* ^*-/-*^ achieved by loss of MDA5 was qualitatively better than MAVS deficiency at the day of birth [17]. A possible explanation may be that there is a difference between the cellular consequences of being *Mavs* ^*-/-*^ and *Ifih1* ^*-/-*^. In *Adar1* ^*-/-*^ *Mavs* ^*-/-*^ and *Adar1p150* ^*-/-*^ *Mavs* ^*-/-*^ cells, unedited endogenous dsRNA – the presumptive candidate immunogenic substrate – are formed and can be sensed by MDA5. The loss of MAVS prevents downstream signaling, but sensing and filament formation by MDA5 remains intact. While the consequences of MDA5 filament formation in the absence of signaling is unknown, it is possible based on the rescue of the *Adar1* ^*E861A/E861A*^ *Ifih1* ^*-/-*^ animals and cells that these may in part contribute to the phenotypes observed. Supporting this, in a cell-based assay of MDA5 function, 5–10% of signaling activity remained in the absence of MAVS [58].

To directly assess the contribution of editing-dependent and editing-independent function of ADAR1 under identical conditions, we assessed pups at the day of birth and used an adult somatic deletion model that leaves animals acutely as either *Adar1*-null (*Adar1* ^*fl/fl*^) or editing deficient (*Adar1* ^*fl/E861A*^). The pups assessed on the day of birth were grossly normal, had normal B cell frequency in the spleen, and no evidence of activated ISGs, indicating that an absence of B cells is not an MDA5-independent function of ADAR1 at the age assessed but may be a phenotype restricted to those rare animals that survived 10–20 days after birth [17]. How these rare *Adar1* ^*-/-*^ *Mavs* ^*-/-*^ animals adapted and survived is unclear. ADAR1p150 appears to have a more specific function in B-cell homeostasis than ADAR1p110 based on the analysis of *Adar1p150* ^*-/-*^ *Mavs* ^*-/-*^, which would not be appreciable in the assays we have completed where both ADAR1 isoforms are either null or editing deficient [17]. In the somatic deletion model that we have applied, there was no/little difference across nearly all cell populations enumerated between the being ADAR1 null and editing-deficient on either an MDA5 WT and null background. This demonstrated that the *Adar1*-null animals were no worse off than animals expressing only an editing-deficient protein. Second, the phenotypes of ADAR1 deficiency or of editing loss were comparably rescued by MDA5 deletion. The somatic deletion of *Adar1* on a *Mavs* ^*-/-*^ background resulted in disrupted intestinal homeostasis five days after tamoxifen treatment [17], with the caveat that the *Adar1* ^*+/+*^ *Mavs* ^*-/-*^ control was not described. This was not observed in the experiments that we completed using the adult deletion model on an *Ifih1* ^*-/-*^ background; however, these experiments have several important differences including Cre strain used and route of tamoxifen administration that limit direct comparison.

While acknowledging there may be editing-independent functions of ADAR1 that are only apparent in early postnatal development, we favor an alternative interpretation. We hypothesize a critically sensitive time point in early post-natal development where unedited dsRNA loads reach a high enough level, akin to the threshold model proposed for DNA sensing [59], that development cannot proceed and where having an ADAR1 protein, even editing-deficient, that can bind RNA is sufficient to “sequester” the dsRNA, can block/reduce signaling to a tolerated level. Whether expression of an editing-deficient cytosolic isoform of ADAR1 would elicit the same effect requires testing. If it were to reduce innate immune signaling in the absence of editing, it would support the concept as has been observed in *C. elegans* and in murine embryonic fibroblasts (MEFs) [26, 60]. The subtle innate immune activation, at least on a transcriptional level in the *Adar1* ^*E861A/E861A*^ *Ifih1* ^*-/-*^ tissues, alludes to alternative minor pathways able to induce the production of ISGs independent of MDA5 (Fig. 2a). Very recently it was reported that Ribonuclease L deficiency could rescue cell death associated with deletion of ADAR1 in a human cell line model [61]. The murine genetic data and rescue indicate that such a mechanism does not get activated in the *Adar1* ^*E861A/E861A*^ *Ifih1* ^*-/-*^ animals, consistent with the concept that having a protein capable of binding RNA, even if incapable of editing, is advantageous compared to being completely deficient for ADAR1.

The present data demonstrate that once MDA5 is neutralized, there are no essential ADAR1-mediated editing events required for mammalian development and adult homeostasis. We do not find evidence for an analogous essential protein recoding event(s) mediated by ADAR1 to that of *Gria2* for ADAR2. Moreover, A-to-I editing by ADAR1 of a variety of substrates, including the editing of miRNAs and their target sequences, outside of modifying the potential for these to interact with MDA5, is neither essential nor required for normal mammalian homeostasis. The parallels between the ADAR1 null and editing-deficient models suggest that editing independent roles of ADAR1 are not significant contributors to the in vivo phenotypes associated with loss of ADAR1. As the transcriptional response and cellular consequences of loss of ADAR1 are conserved across mammals, these data have implications for humans with *ADAR1* mutations, particularly those with AGS [17, 21, 52]. Our data demonstrate that if MDA5 activity/expression can be prevented or reduced, even in the context of a completely editing-deficient ADAR1 protein, this is sufficient to result in largely normal development with limited consequences on long-term organ and tissue homeostasis.

## Conclusions

These analyses demonstrate that the lifetime absence of ADAR1-editing is well tolerated, once MDA5 is inactivated. We conclude that protein recoding arising from ADAR1-mediated editing, unlike ADAR2, is not essential for organismal homeostasis. The comparison of both germline and acute somatic deficient *Adar1* ^*-/-*^ and *Adar1* ^*E861A*^ animals, on either a WT and MDA5-deficient background, did not identify distinct differences between being ADAR1 protein-deficient and having only the expression of an editing dead protein. The phenotypes associated with loss of ADAR1 are the result of RNA editing and MDA5-dependent functions. A-to-I editing by ADAR1 of a variety of substrates, including the editing of miRNAs and their target sequences, outside of modifying the potential for these to interact with MDA5, is neither essential nor required for normal mammalian homeostasis.

## Methods

### Animals

All animal experiments were approved by the AEC (AEC#030/14 and AEC#031/15; St. Vincent’s Hospital, Melbourne). *Adar* ^*E861A/+*^ (*Adar1* ^*E861A/+*^; MGI allele: *Adar* ^*tm1.1Xen*^; MGI:5805648), *Ifih1* ^-/-^ (*Ifih1* ^*tm1.1Cln*^), *Adar* ^*-/-*^ (*Adar1* ^*-/-*^; MGI allele: *Adar* ^*tm2Phs*^; MGI:3029862), *Adar* ^*fl/fl*^ (*Adar1* ^*fl/fl*^; MGI allele: *Adar* ^*tm1.1Phs*^; MGI:3828307), and *Rosa26*-CreER^T2^ (Gt(ROSA)26Sor^tm1(cre/ERT2)Tyj^) mice were on a backcrossed C57BL/6 background as previously described [14, 18, 19, 62, 63]. For day of birth analysis, females were plug mated and pups collected before midday on the day of birth. For acute somatic deletion model, all animals were aged ≥ 8 weeks at tamoxifen initiation; tamoxifen-containing food was prepared at 400 mg/kg tamoxifen citrate (Sigma) in standard mouse chow (Specialty Feeds, WA, Australia).

### Histology

Four (two males, two females) 12-week-old *Adar1* ^*E861A/+*^ *Ifih1* ^*-/-*^ and *Adar1* ^*E861A/E861A*^ *Ifih1* ^*-/-*^ littermates were used for histopathology examination. Tissue collection and histology was performed by the Australian Phenomics Network Histopathology and Organ Pathology Core, University of Melbourne. One male and one female *Adar1* ^*E861A/+*^ *Ifih1* ^*-/-*^ were genotype identified to the pathologists as “controls,” the remaining samples were genotype blinded. Sections were assessed by independent pathologists. The full pathology report is available in Additional file 3: Dataset S1.

### Flow cytometry analysis and fluorescent activated cell sorting

Peripheral blood was analyzed on a hematological analyzer (Sysmex KX-21 N, Roche Diagnostics). Single-cell BM, spleen, and thymus suspensions were prepared by passing through a 23-G needle (BM) or crushing through a 40-μm cell strainer (spleen/thymus) [64]. Antibodies against murine Ter119, CD71, B220, IgM, CD11b/Mac1, Gr1, F4/80, CD43, CD19, CD4, CD8, CD44, Sca-1, c-Kit, CD34, FLT3, FcγR (CD16/32), CD41, CD105, and CD150 were either biotinylated or conjugated with FITC; phycoerythrin, phycoerythrin-Cy5, peridinin chlorophyll protein-Cy5.5, phycoerythrin-Cy7, allophycocyanin, or allophycocyanin eFluor780 were all obtained from eBioscience. CD105 and CD150 were from BioLegend. Biotinylated antibodies were detected with streptavidin conjugated with Brilliant Violet-605 (Biolegend). Cells were analyzed on a BD LSRIIFortessa (BD Biosciences). Results were analyzed with FlowJo software Version 10.0 (Treestar).

For serum cytokine/chemokine expression analysis, serum was isolated from peripheral blood and assessed using a custom bead-based immunoassay (LEGENDPlex, BioLegend) measuring murine IFNγ IFNβ, IL-10, TNFα, CXCL10, CXCL1, CCL2, and CCL11. Serum from age- and sex-matched WT C57B6/J mice from the same rooms of the BioResources Facility were run in parallel. Samples were assessed on BD LSRIIFortessa (BD Biosciences). Results were analyzed with LEGENDPlex software version 7.0 (Biolegend). As a control, tissue culture media was isolated from immortalized myeloid progenitor cells generated from *Rosa26*-CreER^T2^ *Adar1* ^*E861A/fl*^ cell lines that were treated with 200 nM tamoxifen for four days to delete the WT *Adar1* allele. These cells were immortalized with HoxA9 and selected for proliferation in GM-CSF conditioned media as described [44].

### Micro-computed tomography analysis of bone parameters (microCT)

Ex vivo microCT was performed on tibiae using the SkyScan1076 system (Bruker-microCT, Kontich, Belgium) essentially as previously described [47]. Male and female 12-week-old tibia were isolated, fixed overnight in 2% paraformaldehyde, and then stored in 70% ethanol until imaged. Images were acquired using the following settings: pixel size = 9 μm, aluminum filter = 0.5 mm, voltage = 45 kV, current = 220 μA, rotation = 0.7°, and frame averaging = 1. Images were reconstructed and analyzed with SkyScan software programs NRecon (version 1.6.3.3), DataViewer (version 1.4.4), CT Analyser (CTan, version 1.12.0.0), and CTVox (version 2.2.0). The trabecular analysis region of interest (ROI) was determined by identifying the start of the mineralized zone of the proximal growth plate and calculating 3% of the total tibial length towards the tibial mid-shaft, where we then analyzed an ROI of 13.5% of the total tibial length. Analysis of trabecular bone structure was completed using adaptive thresholding (mean of min and max values) in CTan with threshold set at 45–255 for trabecular bone. Cortical analyses were performed at 40% of the total tibial length distal from the mineralized zone of the proximal growth plate and extending for 13.5% of the total tibial length; the threshold values for cortical bone were set to 79–255 and global thresholding algorithm was used. The three-dimensional visualization of trabecular and cortical bone was performed with CTVox, where volume-rendered images were pseudo-colored based on grayscale (pixel) intensity that is reflective of bone mineralization.

### qRT-PCR

Mouse tissues from three independent biological replicates from age-matched and sex-matched C57BL/6, *Adar1* ^*+/+*^ *Ifih1* ^*-/-*^, and *Adar1* ^*E861A/E861A*^ *Ifih1* ^*-/-*^ mice were homogenized in Trisure reagent using IKA T10 basic S5 Ultra-turrax Disperser. Brain tissue was isolated from the pups collected at the day of birth and snap-frozen, then homogenized in Trisure reagent using IKA T10 basic S5 Ultra-turrax Disperser. RNA was extracted using Direct-Zol columns (Zymo Research) as per manufacturer’s instructions. Complementary DNA (cDNA) was synthesized using Tetro cDNA synthesis kit (Bioline). Real-time PCR was done in duplicate with Brilliant II SYBR Green QPCR Master Mix (Agilent Technologies) and primers from IDT (Additional file 3: Table S2). All primers were optimized to have equal efficiency (100+/-10%) before use. *Ppia* was used as a reference gene for relative quantification using the ∆Ct method. RNA was obtained from fetal brain from E12.5 *Adar1* ^*E861A/E861A*^ embryos and processed as described above [19].

### Microfluidics-based multiplex PCR and deep sequencing (mmPCR-seq) identification of A-to-I editing sites

Whole brain, kidney, spleen, thymus, BM, heart, and small intestines from three independent biological replicates from *Adar1* ^*+/+*^ *Ifih1* ^*-/-*^ and *Adar1* ^*E861A/E861A*^ *Ifih1* ^*-/-*^ were used for mmPCR-seq. Following tissue homogenization, RNA was isolated using TRIsure reagent (Bioline) and Direct-Zol columns (Zymo Research). cDNA was synthesized using iScript Advanced Kit (BioRad) using 1.2–7.5 μg RNA per tissue. A total of 200 ng of cDNA was used for mmPCR-seq targeting 557 loci containing 11,103 known A-to-I editing sites [48]. A-to-I editing frequency was assessed using the criteria: minimum coverage > 100 (in 2/3) of WT (*Adar1* ^*+/+*^ *Ifih1* ^*-/-*^) and deficient (*Adar1* ^*E861A/E861A*^ *Ifih1* ^*-/-*^). For a given site, where a replicate had ≤ 100 reads that measurement is regarded as a missing value. All editing sites with editing ≤ 0.01 were removed, leaving 413–808 sites per tissue. Sites were ADAR1-specific targets if the editing level measurements between the WT (*Adar1* ^*+/+*^ *Ifih1* ^*-/-*^) replicates and the editing-deficient (*Adar1* ^*E861A/E861A*^ *Ifih1* ^*-/-*^) replicates were significantly different (*P* < 0.1, ANOVA) and the average editing levels between WT and editing-deficient samples differed by at least 5% [48].

### RNA-seq samples: adult brain

Total RNA was isolated from the whole brain from 12-week-old male *Adar1* ^*+/+*^ *Ifih1* ^*-/-*^ and *Adar1* ^*E861A/E861A*^ *Ifih1* ^*-/-*^ mice (n = 3/genotype). Sequins were included before ribosome depletion [65]. Post ribosome-depleted RNA was purified and subjected to indexing and library preparation using the Kapa Stranded RNA-seq Library Preparation Kit (Kapa Biosystems) and sequenced using the Illumina NextSeq500 with 75-bp paired-end reads by the Ramaciotti Centre for Genomics (UNSW, Australia).

### RNA-seq samples: fetal brain

Total RNA was isolated from the whole brain from E12.5 old male *Adar1* ^*+/+*^ *Ifih1* ^*-/-*^ and *Adar1* ^*E861A/E861A*^ *Ifih1* ^*-/-*^ mice (n = 3/genotype). RNA was purified and subjected to indexing and library preparation using the Illumina TruSeq RNA Sample Prep Kit v2. The libraries were sequenced using the Illumina HiSeq2000 with 100-bp paired-end reads by the Ramaciotti Centre for Genomics (UNSW, Australia).

### RNA-seq analysis

RNA-seq reads from adult brain were aligned to the MM9/NCBIM37 reference genome with STAR [66] and gene counts were determined using “--quantMode GeneCounts.” Differential gene expression analysis was performed using the Degust analysis tool (http://victorian-bioinformatics-consortium.github.io/degust/); briefly, genes were only considered with a CPM of ≥ 2 in 3/3 replicates of either the *Adar1* ^*E861A/E861A*^ *Ifih1* ^*-/-*^ or *Adar1* ^*+/+*^ *Ifih1* ^*-/-*^ (13,891 genes). Normalized read counts (moderated log counts per million) and differential expression were generated using edgeR [67] and genes were considered differentially expressed if FDR < 0.05 (124 genes: 81 up, 43 down). Adding a magnitude requirement to the fold change of abs(log_2_FC) > 1 resulted in 29 genes (24 up, 5 down).

RNA-seq reads from fetal brain were aligned to the MM9/NCBIM37 reference genome with STAR [66] and gene counts were determined using “--quantMode GeneCounts.” Differential gene expression analysis was performed using the Degust analysis tool (http://victorian-bioinformatics-consortium.github.io/degust/); briefly, genes were only considered with a CPM of ≥ 2 in 3/3 replicates of either the *Adar1* ^*E861A/E861A*^ *Ifih1* ^*-/-*^ or *Adar1* ^*+/+*^ *Ifih1* ^*-/-*^ (13,786 genes). Normalized read counts (moderated log counts per million) and differential expression were generated using edgeR [67] and genes were considered differentially expressed if FDR < 0.05 (808 genes: 745 up, 57 down). Adding a magnitude requirement to the fold change of abs(log_2_FC) > 1 resulted in 303 genes (294 up, 9 down).

### GO term enrichment of genes using PANTHER

PANTHER Overrepresentation Test [68] (release 20160715): PANTHER version 11.1 was used to perform statistical testing on sets of differential genes using all *Mus musculus* genes as the reference list against GO biological processes, using Bonferroni multiple testing correction.

### QuSAGE gene set testing

Quantitative Set Analysis for Gene Expression (QuSAGE) was performed as described [69] against the MSigDB collection “C2:curated gene sets” (c2.cgp.v4.0).

### Datasets

All datasets related to this work are deposited in GEO.

RNA-seq accessions:

GSE94387 (adult brain)

GSE94388 (fetal brain)

GSE94386 (mmPCR-seq: BM, spleen, thymus, liver, brain, kidney, small intestine, and heart)

### Statistical analysis

For biological experiments, the significance of results was analyzed using the one-way or two-way ANOVA with multiple comparison corrections unless otherwise stated, *P* < 0.05 was considered significant. All data are presented as mean ± SEM.

## Additional files

Additional file 1: **Movie S1** Video of 12-week-old male mice. (MOV 11186 kb)

Additional file 2: **Movie S2** Video of 12-week-old female mice. (MOV 6740 kb)

Additional file 3: **Dataset S1** Full histopathology report (related to Fig. 4), **Figure S1** (related to Fig. 4) 12-week-old female mouse bone parameters (full legend with figure). **Figure S2** (related to Fig. 7) Spleen and thymus data from *R26*-CreER *Adar1* ^*fl/fl*^ *Ifih1* ^*-/-*^ and *R26*-CreER *Adar1* ^*fl/E861A*^ *Ifih1* ^*-/-*^ and control animals (full legend with figure). **Table S2** qPCR primers used in this study. (PDF 882 kb)

Additional file 4: **Dataset S2** RNA-seq data (related to Fig. 5). (XLSX 9999 kb)

Additional file 5: **Dataset S3** mmPCR-seq data (related to Fig. 6). (XLSX 1061 kb)

Additional file 6: **Table S1** Full statistical comparison of data in Fig. 7. (XLSX 120 kb)
