# Supplementary material for: Protein recoding by ADAR1-mediated RNA editing is not essential for normal development and homeostasis
Source: Genome Biol. 2017 Sep 5;18:166. doi: 10.1186/s13059-017-1301-4 (PMC5585977; doi:10.1186/s13059-017-1301-4)
Supplement: Supplementary file 3 — Dataset S1 Full histopathology report (related to Fig. 4), Figure S1 (related to Fig. 4) 12-week-old female mouse bone parameters (full legend with figure). Figure S2 (related to Fig. 7) Spleen and thymus data from R26-CreER Adar1 fl/fl Ifih1 -/- and R26-CreER Adar1 fl/E861A Ifih1 -/- and control animals (full legend with figure). Table S2 qPCR primers used in this study. (PDF 882 kb) [file 13059_2017_1301_MOESM3_ESM.pdf]

Additional Files:

Additional File 1: Movie S1.mov – Video of 12 week old male mice free on cage. Male 12-week old mice, two *Adar1*$^{E861A/+}$*Ifih1*$^{-/-}$ and one *Adar1*$^{E861A/E861A}$*Ifih1*$^{-/-}$.

Additional File 2: Movie S2.mov – Video of 12 week old female mice free on cage. Female 12-week old mice, two *Adar1*$^{E861A/+}$*Ifih1*$^{-/-}$ and two *Adar1*$^{E861A/E861A}$*Ifih1*$^{-/-}$.

Additional File 3: Dataset S1.pdf – Full histopathology report (related to Figure 4)

Additional File 3: Figure S1.pdf (related to Figure 4) – 12-week old female mouse bone parameters (full legend with figure).

Additional File 3: Figure S2.pdf (related to Figure 7) – Spleen and thymus data from *R26*-CreER *Adar1*$^{fl/fl}$*Ifih1*$^{-/-}$ and *R26*-CreER *Adar1*$^{fl/E861A}$*Ifih1*$^{-/-}$ and control animals (full legend with figure).

Additional File 3: Table S2.pdf – qPCR primers used in this study

Additional File 4: Dataset S2.xls – RNA-seq data (related to Figure 5). *Adar1*$^{E861A/E861A}$*Ifih1*$^{-/-}$ and *Adar1*$^{+/+}$*Ifih1*$^{-/-}$ 12-week old brain count data and differential expression. *Adar1*$^{E861A/E861A}$ and *Adar1*$^{+/+}$ fetal (E12.5) brain count data and differential expression. QuSAGE analysis from adult and fetal brain. PANTHER GO term analysis from adult brain (both combined and separated by up- and down-regulated genes) with multiple expression and logFC cutoffs.

Additional File 5: Dataset S3.xls – mmPCR-seq data and additional analysis of conserved editing sites and mouse ENCODE expression of the transcripts in the indicated tissues (related to Figure 6).

Additional File 6: Table S1.xls – Full statistical comparison of data in Figure 7

Additional file 3: Dataset S1 - full histopathology report

Contacts:

Australian Phenomics Network
Histopathology and Organ Pathology
The University of Melbourne
Department of Anatomy and Neuroscience
Grattan Street, PARKVILLE, VIC 3010

9.1 Histopathology Report

Case Number APN16/010 St. Vincent's Institute of Medical Research (Dr Carl Walkley & Dr Jacki Hearud-Farlow)

Registration Date Mon 29/02/2016

Animal Details

380 (genotype = Adar1 E861A/+ Ifih1-/-)
DOB: 12-15/12/15, 14 weeks, Male, 30.0g, Black
Strain: Mus musculus/C57BL/6/Adar1E861A MDA5

388 (genotype = Adar1 E861A/+ Ifih1-/-)
DOB: 12-15/12/15, 14 weeks, Female, 21.0g, Black
Strain: Mus musculus/C57BL/6/Adar1E861A MDA5

378 (genotype = Adar1 E861A/E861A Ifih1-/-)
DOB: 12-15/12/15, 14 weeks, Male, 25.0g, Black
Strain: Mus musculus/C57BL/6/Adar1E861A MDA5

381 (genotype = Adar1 E861A/+ Ifih1-/-)
DOB: 12-15/12/15, 14 weeks, Female, 23.9g, Black
Strain: Mus musculus/C57BL/6/Adar1E861A MDA5

383 (genotype = Adar1 E861A/E861A Ifih1-/-)
DOB: 12-15/12/15, 14 weeks, Male, 25.1g, Black
Strain: Mus musculus/C57BL/6/Adar1E861A MDA5

385 (genotype = Adar1 E861A/+ Ifih1-/-)
DOB: 12-15/12/15, 14 weeks, Male, 27.8g, Black
Strain: Mus musculus/C57BL/6/Adar1E861A MDA5

386 (genotype = Adar1 E861A/E861A Ifih1-/-)
DOB: 12-15/12/15, 14 weeks, Female, 20.0g, Black
Strain: Mus musculus/C57BL/6/Adar1E861A MDA5

389 (genotype = Adar1 E861A/E861A Ifih1-/-)
DOB: 12-15/12/15, 14 weeks, Female, 20.8g, Black
Strain: Mus musculus/C57BL/6/Adar1E861A MDA5

DoD / Necropsy Tue 15/03/2016

Origin St. Vincent's Institute of Medical Research

Treatment Treatment
Whole live animals - apparently normal and healthy.
#380 and 388 respectively are examples of control for males and females.
DOB12-15/12/15
(C. Walkley)

Species / Breed / Strain Mus musculus/C57BL/6/Adar1E861A MDA5

**Animal Health Facility**	St. Vincent's Bioresources Centre<br>POSITIVE for Mouse Norovirus POSITIVE<br>for Helicobacter hepaticus<br>POSITIVE for Helicobacter spp<br>POSITIVE for Helicobacter typhlonius<br>POSITIVE for Pasteurella pneumotropica<br>POSITIVE for Pasteurellaceae spp (not including pneumotropica)<br>POSITIVE for Chilomastix bettencourti<br>POSITIVE for Entamoeba muris
**Organs Examined**	Adrenal glands, Bladder, Bone marrow, Brain, Cecum, Cervix, Clitoral gland, Colon, Duodenum, Epididymes, Eyes, Gall bladder, Harderian glands, Head, Heart, Hind leg (Long bone, Bone marrow, Synovial joint, Skeletal muscle), Ileum, Jejunum, Kidney, Liver, Lungs, Mammary tissue, Mesenteric lymph node, Other tissue, Ovaries, Oviducts, Pancreas, Penis, Preputial gland, Prostate glands, Salivary glands and Regional lymph nodes, Seminal vesicles, Skin, Spinal cord, Spleen, Stomach, Tail, Testes, Thymus, Thyroids, Trachea, Uterus, Vagina
**Macroscopic Observations**	Delivered to the APN Tuesday AM 15th March.<br><br>At the time of necropsy, the animals appeared well nourished, well groomed, active/curious and healthy with normal movement and gait. There were no observable dermal lesions and no nasal/ocular discharges. The gastrointestinal tract contained ample ingesta and the thoracic and abdominal viscera showed no macroscopic abnormalities. Mildly distended intestine in animal #383.<br><br>Note:<br>(i)Reactive lymph nodes are defined as mild follicular hyperplasia, germinal centre formation and occasional sinus histiocytosis- a common finding in mice<br>(ii) Mild extramedullary hematopoiesis (EMH) identified in the red pulp of all the spleens, a common finding in the mouse. EMH consists of erythroid precursors, myeloid precursors, megakaryocytes or all three. While some degree of extramedullary hematopoiesis is present in normal rodents, especially in mice, increased extramedullary hematopoiesis can result from hematotoxic insult, systemic anemia, and infections elsewhere in the body.<br><br>(iii)Sexual dimorphism, under the influence of testosterone exists in the submandibular glands of the mouse. The male glands are larger and weigh almost twice as much as those of the female. In the male, the convoluted (granular) ducts are larger and more prominent than the female, and are lined by large, tall columnar cells, containg abundant eosinophilic intra-cytoplasmic granules. In the female , these ducts are lined by shorter columnar cells with centrally located nuclei and fewer intra-cytoplasmic granules.

Microscopic Observations

Blood counts: Most readings are within the normal mouse reference intervals. The Hematocrit was elevated (this is likely to be a result of mild dehydration as the RBC count was normal). The platelet count for most samples including the controls was low, worth noting as the MPV was elevated in some cases indicating the presence of large, immature platelets. Please note that mouse platelets readily aggregate and instrument generated platelet counts will underestimate true platelet counts in the presence of platelet clumping. In addition, mouse platelet clumps are counted as eosinophils by some automated hematology analyser. Note that the eosinophil count for all samples was elevated.

380 (control)

Macro Observations

Testes: 5x4x3mm, symmetrical
Spleen: 14x4x3mm
Kidneys: 12x7x5mm, symmetrical
Thymus: 10x9x2mm
Heart: 10x7x6mm
Brain: 15x11x6mm, symmetrical
Pituitary gland identified, macroscopically normal
Tail 80mm (straight)
Teeth, tongue and oral mucosa unremarkable - Head harvested for evaluation of auditory and vestibular structures
Tail suspension test for neurological defects-negative
Left hind leg-Bone marrow smear

Ears harvested and frozen
Left tibia collected and fixed overnight in 10% NBF at room temperature then transferred to 70% ethanol

Micro Observations

Peripheral blood smear: Examination of the smear showed red blood cells (majority of cells shown), occasional white blood cells including segmented neutrophils and platelets (clumps). No discernible morphological changes or detectable parasites (52853)

Marrow smear: Cells observed were readily identified as those from the erythroid and myeloid series. Conspicuous cells from the lymphoid series. Discernible and unremarkable megakaryoblasts (52854).

#380 was used as a male histological control

Testes- Occasional seminiferous tubule with mild testicular degeneration
Cecum- Numerous intraluminal protozoa

388 (control)

Macro Observations

Spleen: 12x4x2mm
Kidneys: 10x6x5, symmetrical
Thymus: 5x7x2mm
Heart: 8x6x5mm
Brain: 15x10x5mm, symmetrical
Pituitary gland identified, macroscopically normal
Tail 80mm (straight)
Teeth, tongue and oral mucosa unremarkable - Head harvested for evaluation of auditory and vestibular structures
Tail suspension test for neurological defects-negative
Left hind leg-Bone marrow smear

A single ear was harvested and frozen
Left tibia & femur collected and fixed overnight in 10% NBF at room temperature then transferred to 70% ethanol

Micro Observations

Peripheral blood smear: Examination of the smear showed red blood cells (majority of cells shown), occasional white blood cells including segmented neutrophils and platelets (clumps). No discernible morphological changes or detectable parasites (52864)

Marrow smear: Cells observed were readily identified as those from the erythroid and myeloid series. Conspicuous cells from the lymphoid series. Discernible and unremarkable

megakaryoblasts (52863).

#388 was used as a female histological control

Cecum- Numerous intraluminal protozoa

378

Macro Observations

Testes: 6x5x4mm, symmetrical
Spleen: 15x4x2mm
Kidneys: 12x6x5mm, symmetrical
Thymus: 8x7x2mm
Heart: 10x7x6mm
Brain: 15x10x5mm, symmetrical
Pituitary gland identified, macroscopically normal
Tail 80mm (straight)
Teeth, tongue and oral mucosa unremarkable - Head harvested for evaluation of auditory and vestibular structures
Tail suspension test for neurological defects-negative
Left hind leg-Bone marrow smear

Ears harvested and frozen
Left tibia collected and fixed overnight in 10% NBF at room temperature then transferred to 70% ethanol

Micro Observations

Peripheral blood smear: Examination of the smear showed red blood cells (majority of cells shown), occasional white blood cells including segmented neutrophils and platelets (clumps). No discernible morphological changes or detectable parasites (52851)

Marrow smear: Cells observed were readily identified as those from the erythroid and myeloid series. Conspicuous cells from the lymphoid series. Discernible and unremarkable megakaryoblasts (52852).

Testes/Epididymes

Section shows typical convoluted seminiferous tubules at various stages of cycle surrounded by the tunica albuginea. Within the tubules, unremarkable spermatogenic cells including, Sertoli cells, spermatogonia, developing spermatocytes and spermatids. Section also shows unremarkable vas deferens with typical intraluminal sperm.
The architecture of the epididymis is typical, with numerous intraluminal elongated spermatozoa.

No lesions of significance
(52689)

Seminal vesicles

Unremarkable tall columnar epithelium and folded mucosa
Presence of typical intraluminal eosinophilic secretions

No lesions of significance
(52691)

Prostate glands

Section shows unremarkable ventral/coagulating prostate glands with typical intraluminal secretions.
Section shows concretions in the prostatic urethra, commonly seen as an incidental finding
No lesions of significance
(52691)

Penis/Preputial gland

Section shows typical penile structures including prepuce, glans, corpus cavernosum and urethra.
Unremarkable preputial glands including basal and secretory cells.

No lesions of significance
(52698)

Urinary Bladder

Unremarkable distended bladder with typical urothelium and detrusor muscle.

No lesions of significance
(52691)

Liver/Gall bladder

Section shows typical liver parenchyma including hepatocytes, Kupffer cells, portal triads and central veins.
Unremarkable Gall bladder

No lesions of significance
(52696)

Stomach

Section show fore and glandular portions of the stomach with limiting ridge. In the glandular portion, mild enterocyte hyperplasia, mild inflammation (neutrophils) in the lower gastric glands extending into the underlying muscularis.
(52687)

Comments:

Pathology to comment

Small Intestine (Duodenum, Jejunum & Ileum)/GALT

Section shows typical mucosal villi and submucosal layers. Mild enterocyte hyperplasia and prominent mitotic figures.
Peyer’s patches displayed typical reactive nodal histology.
(52702, 52706)

Comments:

Pathology to comment

Cecum/Colon/GALT

Typical mucosal folds and submucosal layers and typical lymphoid clusters (Peyer's patch) with reactive micromorphology
Marked intraluminal protozoan infestation in the cecum.
(52703, 52704)

Mesenteric lymph node

Section shows small portion of mesenteric lymph node with typical nodal histology including cortex, medullary cords and sinuses.

No lesions of significance
(52695)

Spleen

Unremarkable follicular pattern identified with typical red and white pulp micromorphology.
Mild extramedullary hematopoiesis identified in the red pulp of the spleen, a common finding in the mouse.

No lesions of significance
(52692)

Pancreas

Section shows representative exocrine tissue (serous acini) and endocrine tissue (islets of Langerhans).

No lesions of significance
(52692)

Kidney

Section shows a cortex, medulla, and papilla. There is a uniform distribution of Glomeruli and accompanying nephron components and the micromorphology of the convoluted and straight segment tubules was unremarkable.
Small clusters of tubular casts were observed in the cortex.
These micromorphological changes were considered mild.

(52701)

Comments:

Pathology to comment

Adrenal glands

Section shows adrenal glands with typical cortex/medulla micromorphology.

No lesions of significance
(52701)

Salivary glands and Regional lymph nodes

Section shows submandibular, sublingual and parotid glands.
The regional lymph nodes displayed mild follicular hyperplasia with germinal centre formation, indicating a reactive state.

No lesions of significance
(52695)

Comments:

Observation: In the submandibular glands, there appears to be a paucity of serous acini compared to the control male

Thyroids

Normal lateral lobes of the thyroid gland with typical follicles lined by cuboidal epithelium.
Section also includes representative parathyroid gland.

No lesions of significance
(52690)

Trachea/Lungs

Section shows typical lung micromorphology demonstrating parenchyma/alveoli, bronchioles, blood vessels and parabronchial lymph node.
Various degrees of interstitial and vascular congestion judged to be artefactual.
Trachea with unremarkable mucosal epithelial lining and hyaline cartilage.
Oesophagus with typical features including stratified squamous epithelium

No lesions of significance
(52690)

Thymus

Section shows typical medulla/cortex distribution and micromorphology.

No lesions of significance
(52688)

Heart/chambers/vessels/valves

Typical micromorphological observed in cardiac muscle, chambers, valves and vessels of the heart.
The cardiac muscle fibres demonstrated typical features including central nuclei, branching fibres and striations.

No lesions of significance
(52700)

Skin

Typical dermal appendages and distribution

No lesions of significance
(52697)

Tail

Section shows typical tail components including keratinized squamous epithelium, dense regular connective tissue, tendons, bone,intervertebral disc, bone marrow, skeletal muscle, nerves and blood vessels.

No lesions of significance
(52694)

Eyes/Harderian glands

Section shows eyes with unremarkable retina, cornea, iris, ciliary body, lens, sclera and choroid.
Typical branched tubuloalveolar formation of the Harderian gland.

No lesions of significance
(52705)

Brain

Sections were prepared from the standard levels of the brain:

Level I: including cortex, corpus callosum, the lateral ventricles and caudate putamen (Bregma -0.46)
Level II: includes the hippocampus, thalamus, amygdala,hypothalamus, lateral and third ventricles (Bregma -2.18)
Level III: includes the cerebellum, pons and fourth ventricle (Bregma -5.40)

Sections of brain stained with Haematoxylin and Eosin, Luxol Fast Blue appear symmetrical with no ventricular dilation observed, unremarkable meninges and typical lamination.
The cerebellum appears symmetrical with typical architecture and Purkinje cells.
At this level, there was no evidence of neuronal loss and the myelination appeared normal.

No lesions of significance
(52707, 52867)

Comments:

Neuropathology to comment

Spinal cord

Representative thoracic and lumbar region of spinal cord, vertebral bone, striated muscle, peripheral nerves and bone marrow.

No lesions of significance
(52693)

(Hind leg) Long bone/Bone marrow/Synovial joint/Skeletal muscle

Levels through the hind leg show unremarkable long bone, striated muscle, synovial joint and bone marrow including conspicuous megakaryoblasts. The skeletal muscle shows consistent fiber size with peripheral nuclei.

No lesions of significance
(52699, 54804)

Head

Multiple levels through the head demonstrate dermal appendages, nasal cavity,oral cavity, teeth and tongue including muscle bundles (52876-77). Sections also show unremarkable pituitary gland including pars intermedia, pars distalis and pars nervosa
as well and the trigeminal nerve/ganglia (52875).The outer and middle regions of the ear are discernible. The tympanic membrane is intact and the ossicles are unremarkable and include the stapedial annular ligaments (52876-77).
Typical components of the inner ear including bony labyrinth, organ of corti, stria vascularis and scala cavities are discernible. Based on multiple levels, the organ of corti is unremarkable with no discernible loss of inner/outer hair cells and typical tectorial membrane (52876-77).
The cochlear nerve and spiral ganglion is also demonstrated and based on several levels, there is no reduction in the density of the spiral ganglion cells. Examples of otolith organs can be seen with typical features such as the hair cells and mineral otoliths. The ampulla including the crista ridge with hair cells is discernible (52878)

No lesions of significance
(52875-79)

381

Macro Observations

Spleen: 14x4x2mm
Kidneys: 11x6x5mm, symmetrical

Thymus: 10x8x2mm
Heart: 9x6x6mm
Brain: 14x11x6mm, symmetrical
Pituitary gland identified, macroscopically normal
Tail 85mm (straight)
Teeth, tongue and oral mucosa unremarkable - Head harvested for evaluation of auditory and vestibular structures
Tail suspension test for neurological defects-negative
Left hind leg-Bone marrow smear

Ears harvested and frozen
Left tibia collected and fixed overnight in 10% NBF at room temperature then transferred to 70% ethanol

Micro Observations

Peripheral blood smear: Examination of the smear showed red blood cells (majority of cells shown), numerous white blood cells including segmented neutrophils and platelets (clumps). No discernible morphological changes or detectable parasites (52859)

Marrow smear: Cells observed were readily identified as those from the erythroid and myeloid series. Conspicuous cells from the lymphoid series. Discernible and unremarkable megakaryoblasts (52860).

Mammary glands

Section shows typical mammary fat pad with developing lactiferous ducts (including primary duct), blood vessels, nerve
bundles and a large reactive lymph node.

No lesions of significance
(52787)

Ovaries/Oviducts

Section shows unremarkable ovaries containing follicles at various stages of development and several corpora lutea. Unremarkable oviduct micromorphology with typical columnar epithelium and mucosal folds.

No lesions of significance
(52784)

Uterus/Cervix/Vagina/Clitoral gland

Unremarkable architecture of the endometrium/ endometrial glands, myometrium and adventitia. The micromorphology of the uterus and vagina places the animal at estrus.

No lesions of significance
(52784)

Urinary Bladder

Unremarkable collapsed bladder with typical urothelium and detrusor muscle

No lesions of significance
(52784)

Liver/Gall bladder

Section shows typical liver parenchyma including hepatocytes, Kupffer cells, portal triads and central veins. Unremarkable Gall bladder.

No lesions of significance
(52786)

Stomach

Section shows unremarkable fore and glandular portions of the stomach with limiting ridge.
Query mild enterocyte hyperplasia
(52789)

Comments:

Pathology to comment

Small Intestine (Duodenum, Jejunum & Ileum)/GALT

Section shows typical mucosal villi and submucosal layers. Mild enterocyte hyperplasia and prominent mitotic figures.
Peyer’s patches displayed typical reactive nodal histology.
(52775, 52779)

Comments:

Pathology to comment

Cecum/Colon/GALT

Typical mucosal folds and submucosal layers and typical lymphoid clusters (Peyer's patch) with reactive micromorphology.
Marked intraluminal protozoan infestation in the cecum, proximal and distal colon.
(52772, 52773)

Comments:

Pathology to comment

Mesenteric lymph node

Section shows typical reactive mesenteric lymph node with representative cortex including the occasional follicle, an expansive paracortical area and mild sinus histiocytosis.

No lesions of significance
(52785)

Spleen

Unremarkable follicular pattern identified with typical red and white pulp micromorphology.
Mild to moderate extramedullary hematopoiesis identified in the red pulp of the spleen.

No lesions of significance
(52783)

Pancreas

Section shows representative exocrine tissue (serous acini) and endocrine tissue (islets of Langerhans).

No lesions of significance
(52783)

Kidney

Section shows typical cortex with a uniform distribution of Glomeruli and accompanying nephron components. The micromorphology of the tubules is unremarkable.

No lesions of significance
(52778)

Adrenal glands

Section shows one adrenal gland with typical cortex/medulla micromorphology.

No lesions of significance
(52778)

Salivary glands and Regional lymph nodes

Section shows unremarkable submandibular and parotid glands as well as brown fat.

No lesions of significance
(52785)

Thyroids

Normal lateral lobes of the thyroid gland with typical follicles lined by cuboidal epithelium.

No lesions of significance
(52788)

Trachea/Lungs

Section shows typical lung micromorphology demonstrating parenchyma/alveoli, bronchioles, blood vessels and parabronchial lymph node. Mild vascular and interstitial congestion judged to be artefactual.
Trachea with unremarkable mucosal epithelial lining and hyaline cartilage.
Oesophagus with typical features including stratified squamous epithelium

No lesions of significance
(52788)

Thymus

Section shows typical medulla/cortex distribution and micromorphology.

No lesions of significance
(52790)

Heart/chambers/vessels/valves

Typical micromorphological observed in cardiac muscle, chambers, valves and vessels of the heart. The cardiac muscle fibres demonstrated typical features including central nuclei, branching fibres and striations.

No lesions of significance
(52777)

Skin

Typical dermal appendages and distribution

No lesions of significance
(52782)

Tail

Section shows typical tail components including keratinized squamous epithelium, dense regular connective tissue, tendons, bone, intervertebral disc, bone marrow, skeletal muscle, nerves and blood vessels.

No lesions of significance
(52781)

Eyes/Harderian glands

Section shows eyes with unremarkable retina, cornea, iris, ciliary body, lens, sclera and choroid. Typical branched tubuloalveolar formation of the Harderian gland.

No lesions of significance
(52774)

Brain

Sections were prepared from the standard levels of the brain:

Level I: including cortex, corpus callosum, the lateral ventricles,anterior commissure and caudate putamen (Bregma -0.98)
Level II: includes the hippocampus, thalamus, amygdala,hypothalamus, lateral and third ventricles (Bregma -1.94)
Level III: includes the cerebellum, pons and fourth ventricle (Bregma -5.34)

Sections of brain stained with Haematoxylin and Eosin, Luxol Fast Blue appear symmetrical with no ventricular dilation observed, unremarkable meninges and typical lamination. The cerebellum appears symmetrical with typical architecture and Purkinje cells. At this level, there was no evidence of neuronal loss and the myelination appears normal.

No lesions of significance
(52771, 52871)

Comments:

Neuropathology to comment

Spinal cord

Representative thoracic and lumbar region of spinal cord, vertebral bone, striated muscle, peripheral nerves and bone marrow.

No lesions of significance
(52780)

Comments:

Neuropathology to comment

(Hind leg) Long bone/Bone marrow/Synovial joint/Skeletal muscle

Levels through the hind leg show unremarkable long bone, striated muscle, synovial joint and bone marrow including conspicuous megakaryoblasts. The skeletal muscle shows consistent fiber size with peripheral nuclei.

No lesions of significance
(52776, 54808)

Head

Multiple levels through the head demonstrate dermal appendages, nasal cavity,oral cavity, teeth and tongue including muscle bundles (52898). Sections also show unremarkable pituitary gland including pars intermedia, pars distalis and pars nervosa
as well and the trigeminal nerve/ganglia (52895).The outer and middle regions of the ear are discernible. The tympanic membrane is intact and the ossicles are unremarkable and include the stapedial annular ligaments (52897, 52898).
Typical components of the inner ear including bony labyrinth, organ of corti, stria vascularis and scala cavities are discernible. Based on multiple levels, the organ of corti is unremarkable with no discernible loss of inner/outer hair cells and typical tectorial membrane (52897).
The cochlear nerve and spiral ganglion is also demonstrated and based on several levels, there is no reduction in the density of the spiral ganglion cells. Examples of otolith organs can be seen

with typical features such as the hair cells and mineral otoliths. The ampulla including the crista ridge with hair cells is discernible (52898)

No lesions of significance
(52895-52899)

383

Macro Observations

Testes: 5x4x4mm, symmetrical
Spleen: 12x5x2mm
Kidneys: 11x6x5mm, symmetrical
Thymus: 7x5x2mm
Heart: 8x6x5mm
Brain: 15x10x5mm, symmetrical
Pituitary gland identified, macroscopically normal
Tail 85mm (straight)
Teeth, tongue and oral mucosa unremarkable - Head harvested for evaluation of auditory and vestibular structures
Tail suspension test for neurological defects-negative
Left hind leg-Bone marrow smear
Note: intestine appeared distended

Ears harvested and frozen
Left tibia collected and fixed overnight in 10% NBF at room temperature then transferred to 70% ethanol

Micro Observations

Peripheral blood smear: Examination of the smear showed red blood cells (majority of cells shown), numerous white blood cells including segmented neutrophils and platelets (clumps). No discernible morphological changes or detectable parasites (52856)

Marrow smear: Cells observed were readily identified as those from the erythroid and myeloid series. Conspicuous cells from the lymphoid series. Discernible and unremarkable megakaryoblasts (52855).

Testes/Epididymes

Section shows typical convoluted seminiferous tubules at various stages of cycle surrounded by the tunica albuginea. Within the tubules, unremarkable spermatogenic cells including, Sertoli cells, spermatogonia, developing spermatocytes and spermatids.
The architecture of the epididymis is typical, with numerous intraluminal elongated spermatozoa.

No lesions of significance
(52745)

Seminal vesicles

Unremarkable tall columnar epithelium and folded mucosa
Presence of typical intraluminal eosinophilic secretions

No lesions of significance
(52738)

Prostate glands

Section shows unremarkable ventral and dorsal lateral prostate glands with typical intraluminal secretions, typical vas deferens and a portion of ureter.
Section shows concretions in the prostatic urethra, commonly seen as an incidental finding
No lesions of significance
(52738)

Penis/Preputial gland

Section shows typical urethra and unremarkable preputial glands including basal and secretory cells.

No lesions of significance
(52740)

Urinary Bladder

Unremarkable distended bladder with typical urothelium and detrusor muscle.

No lesions of significance
(52738)

Liver/Gall bladder

Section shows typical liver parenchyma including hepatocytes, Kupffer cells, portal triads and central veins. Occasionally a small aggregation of Kuppfer cells, probably trivial hepatocyte loss from itinerant gut bacteria, and a common incidental finding.
Unremarkable Gall bladder

No lesions of significance
(52734)

Stomach

Section show fore and glandular portions of the stomach with limiting ridge. In the glandular portion, mild enterocyte hyperplasia, mild inflammation (neutrophils) in the lower gastric glands extending into the underlying muscularis.
(52742)

Comments:

Pathology to comment

Small Intestine (Duodenum, Jejunum & Ileum)/GALT

Section shows typical mucosal villi and submucosal layers. Mild enterocyte hyperplasia/crowding and prominent mitotic figures.
Peyer’s patches displayed typical reactive nodal histology.
(52732, 52736)

Comments:

Pathology to comment

Cecum/Colon/GALT

Typical mucosal folds and submucosal layers and typical lymphoid clusters (Peyer's patch) with reactive micromorphology. Marked intraluminal protozoan infestation in the cecum and entire length of colon.
(52747, 52748)

Comments:

Pathology to comment

Mesenteric lymph node

Section shows small portion of mesenteric lymph node with typical nodal histology including cortex, medullary cords and sinuses.

No lesions of significance
(52733)

Spleen

Unremarkable follicular pattern identified with typical red and white pulp micromorphology. Moderate extramedullary hematopoiesis identified in the red pulp of the spleen.

No lesions of significance
(52737)

Comments:

Pathology to comment

Pancreas

Section shows representative exocrine tissue (serous acini) and endocrine tissue (islets of Langerhans).

No lesions of significance
(52737)

Kidney

Section shows a cortex, medulla with a uniform distribution of Glomeruli and accompanying nephron components. The micromorphology of the tubules is unremarkable.

No lesions of significance
(52735)

Adrenal glands

Section shows one adrenal gland with typical cortex/medulla micromorphology.

No lesions of significance
(52735)

Salivary glands and Regional lymph nodes

Section shows submandibular, sublingual and parotid glands.
The regional lymph nodes show typical nodal histology including cortex, medullary cords and sinuses.

No lesions of significance
(52733)

Comments:

Observation: In the submandibular glands, there is a paucity of serous acini compared to the control male

Thyroids

Unremarkable colloid secreting follicles identified. Section also shows a small sheet-like mass of polygonal cells, characteristic of the parathyroid gland.

No lesions of significance
(52746)

Trachea/Lungs

Section shows typical lung micromorphology demonstrating parenchyma/alveoli, bronchioles, blood vessels and parabronchial lymph node.
Trachea with unremarkable mucosal epithelial lining and hyaline cartilage.
Oesophagus with typical features including stratified squamous epithelium and discernible intraluminal protozoan aggregates.

No lesions of significance
(52746)

Comments:

Pathology to comment

Thymus

Section shows typical medulla/cortex distribution and micromorphology.

No lesions of significance
(52741)

Heart/chambers/vessels/valves

Typical micromorphological observed in cardiac muscle, chambers, valves and vessels of the heart.
The cardiac muscle fibres demonstrated typical features including central nuclei, branching fibres and striations.

No lesions of significance
(52730)

Skin

Typical dermal appendages and distribution

No lesions of significance
(52739)

Tail

Section shows typical tail components including keratinized squamous epithelium, dense regular connective tissue, tendons, bone, bone marrow, skeletal muscle, nerves and blood vessels.

No lesions of significance
(52743)

Eyes/Harderian glands

Section shows eyes with unremarkable retina, cornea, iris, ciliary body, lens, sclera and choroid.
Typical branched tubuloalveolar formation of the Harderian gland.

No lesions of significance
(52731)

Brain

Sections were prepared from the standard levels of the brain:

Level I: including cortex, corpus callosum, the third and lateral ventricles, choroid plexus and caudate putamen (Bregma -0.58)
Level II: includes the hippocampus, thalamus, amygdala,hypothalamus, lateral and third ventricles (Bregma -1.94)
Level III: includes the cerebellum, pons and fourth ventricle (Bregma -6.36)

Sections of brain stained with Haematoxylin and Eosin, Luxol Fast Blue appear symmetrical with no ventricular dilation observed, unremarkable meninges and typical lamination. The cerebellum appears symmetrical with typical architecture and Purkinje cells. At this level, there was no evidence of neuronal loss and the myelination appeared normal.

No lesions of significance
(52749, 52869)

Comments:

Neuropathology to comment

Spinal cord

Representative thoracic and lumbar region of spinal cord, vertebral bone, striated muscle, peripheral nerves and bone marrow.

No lesions of significance
(52744)

Comments:

Neuropathology to comment

(Hind leg) Long bone/Bone marrow/Synovial joint/Skeletal muscle

Levels through the hind leg show unremarkable long bone, striated muscle, synovial joint and bone marrow including conspicuous megakaryoblasts. The skeletal muscle shows consistent fiber size with peripheral nuclei.

No lesions of significance
(52729, 54806)

Head

Multiple levels through the head demonstrate dermal appendages, nasal cavity,oral cavity, teeth and tongue including muscle bundles (52885-52889). Sections also show unremarkable pituitary gland including pars intermedia, pars distalis and pars nervosa
as well and the trigeminal nerve/ganglia (52885).The outer and middle regions of the ear are discernible. The tympanic membrane is intact and the ossicles are unremarkable and include the stapedial annular ligaments (52887-88).
Typical components of the inner ear including bony labyrinth, organ of corti, stria vascularis and scala cavities are discernible. Based on multiple levels, the organ of corti is unremarkable with no discernible loss of inner/outer hair cells and typical tectorial membrane (52887).
The cochlear nerve and spiral ganglion is also demonstrated and based on several levels, there is no reduction in the density of the spiral ganglion cells. Examples of otolith organs can be seen with typical features such as the hair cells and mineral otoliths (52887-88).

No lesions of significance
(52885--89)

385

Macro Observations

Testes: 5x3x3mm, symmetrical
Spleen: 13x4x2mm
Kidneys: 12x6x5mm, symmetrical
Thymus: 8x6x3mm
Heart: 9x6x6mm
Brain: 15x10x6mm, symmetrical
Pituitary gland identified, macroscopically normal
Tail 83mm (straight)

Teeth, tongue and oral mucosa unremarkable - Head harvested for evaluation of auditory and vestibular structures
Tail suspension test for neurological defects-negative
Left hind leg-Bone marrow smear

Ears harvested and frozen
Left tibia collected and fixed overnight in 10% NBF at room temperature then transferred to 70% ethanol

Micro Observations

Peripheral blood smear: Examination of the smear showed red blood cells (majority of cells shown), occasional white blood cells including segmented neutrophils and platelets (clumps). No discernible morphological changes or detectable parasites (52858)

Marrow smear: Cells observed were readily identified as those from the erythroid and myeloid series. Conspicuous cells from the lymphoid series. Discernible and unremarkable megakaryoblasts (52857).

Testes/Epididymes

Section shows typical convoluted seminiferous tubules at various stages of cycle surrounded by the tunica albuginea. Within the tubules, unremarkable spermatogenic cells including, Sertoli cells, spermatogonia, developing spermatocytes and spermatids.
The architecture of the epididymis is typical, with numerous intraluminal elongated spermatozoa.

No lesions of significance
(52754)

Seminal vesicles

Unremarkable tall columnar epithelium and folded mucosa
Presence of typical intraluminal eosinophilic secretions

No lesions of significance
(52770)

Prostate glands

Section shows unremarkable dorsal lateral and coagulating prostate glands with typical intraluminal secretions.

No lesions of significance
(52770)

Penis/Preputial gland

Section shows typical penile structures including prepuce, glans, corpus cavernosum and urethra. Unremarkable preputial glands including basal and secretory cells.

No lesions of significance
(52768)

Urinary Bladder

Unremarkable distended bladder with typical urothelium and detrusor muscle.

No lesions of significance
(52770)

Liver/Gall bladder

Section shows typical liver parenchyma including hepatocytes, Kupffer cells, portal triads and central veins.
Section does not show Gall bladder

No lesions of significance
(52766)

Stomach

Section show fore and glandular portions of the stomach with limiting ridge.
Query mild enterocyte hyperplasia

(52752)

Comments:

Pathology to comment

Small Intestine (Duodenum, Jejunum & Ileum)/GALT

Section shows typical mucosal villi and submucosal layers. Mild enterocyte hyperplasia/crowding and prominent mitotic figures.
Peyer’s patches displayed typical reactive nodal histology.
(52760, 52764)

Comments:

Pathology to comment

Cecum/Colon/GALT

Typical mucosal folds and submucosal layers and typical lymphoid clusters (Peyer's patch) with reactive micromorphology.
Marked intraluminal protozoan infestation in the cecum and proximal colon.
(52758-59)

Comments:

Pathology to comment

Mesenteric lymph node

Section shows mesenteric lymph node with typical reactive micromorphology including mild follicular hyperplasia with germinal centre formation, indicating a reactive state.

No lesions of significance
(52767)

Spleen

Unremarkable follicular pattern identified with typical red and white pulp micromorphology.
Moderate extramedullary hematopoiesis identified in the red pulp of the spleen.

No lesions of significance
(52751)

Comments:

Pathology to comment

Pancreas

Section shows representative exocrine tissue (serous acini) and endocrine tissue (islets of Langerhans).

No lesions of significance
(52751)

Kidney

Section shows a cortex, medulla, and papilla. There is a uniform distribution of Glomeruli and accompanying nephron components and the micromorphology of the convoluted and straight segment tubules was unremarkable.
Mild processing/fixation artefact

No lesions of significance
(52765)

Comments:

Pathology to comment

Adrenal glands

Section shows one adrenal gland with typical cortex/medulla micromorphology.

No lesions of significance
(52765)

Salivary glands and Regional lymph nodes

Section shows submandibular, sublingual and parotid glands.
The regional lymph nodes displayed mild follicular hyperplasia with germinal centre formation, indicating a reactive state.

No lesions of significance
(52767)

Comments:

Observation: Unremarkable serous acini numbers in the submandibular glands compared to the control male.

Thyroids

Unremarkable colloid secreting follicles identified. Section also shows a small sheet-like mass of polygonal cells, characteristic of the parathyroid gland.

No lesions of significance
(52756)

Trachea/Lungs

Section shows typical lung micromorphology demonstrating parenchyma/alveoli, bronchioles, blood vessels and parabronchial lymph node.

Trachea with unremarkable mucosal epithelial lining and hyaline cartilage.
Oesophagus with typical features including stratified squamous epithelium.

No lesions of significance
(52756)

Thymus

Section shows typical medulla/cortex distribution and micromorphology. A small cortical cyst was identified.

(52741)

Comments:

Thymic cysts represent either a dilation of thymic tubular structures or remnants of the thymopharyngeal ducts. Cysts are a common finding in aged mice or they may be associated with epithelial glandular hyperplasia.
Pathology of the Mouse, R.R Maronpot

Pathology to comment

Heart/chambers/vessels/valves

Typical micromorphological observed in cardiac muscle, chambers, valves and vessels of the heart.
The cardiac muscle fibres demonstrated typical features including central nuclei, branching fibres and striations.

No lesions of significance
(52762)

Skin

Typical dermal appendages and distribution

No lesions of significance
(52769)

Tail

Section shows typical tail components including keratinized squamous epithelium, dense regular connective tissue, tendons, bone, bone marrow, skeletal muscle, nerves and blood vessels.

No lesions of significance
(52753)

Eyes/Harderian glands

Section shows eyes with unremarkable retina, cornea, iris, ciliary body, lens, sclera and choroid.
Typical branched tubuloalveolar formation of the Harderian gland.
No lesions of significance
(52761)

Brain

Sections were prepared from the standard levels of the brain:

Level I: including cortex, corpus callosum, the third and lateral ventricles, choroid plexus and caudate putamen (Bregma -0.58)
Level II: includes the hippocampus, thalamus, amygdala,hypothalamus, lateral and third ventricles (Bregma -2.54)
Level III: includes the cerebellum, pons and fourth ventricle (Bregma -6.00)

Sections of brain stained with Haematoxylin and Eosin, Luxol Fast Blue appear symmetrical with no ventricular dilation observed, unremarkable meninges and typical lamination. The cerebellum appears symmetrical with typical architecture and Purkinje cells. At this level, there was no evidence of neuronal loss and the myelination appeared normal.

No lesions of significance

(52757, 52870)

Comments:

Neuropathology to comment

Spinal cord

Representative thoracic and lumbar region of spinal cord, vertebral bone, striated muscle, peripheral nerves and bone marrow.

No lesions of significance
(52750)

Comments:

Neuropathology to comment

(Hind leg) Long bone/Bone marrow/Synovial joint/Skeletal muscle

Levels through the hind leg show unremarkable long bone, striated muscle, synovial joint and bone marrow including conspicuous megakaryoblasts. The skeletal muscle shows consistent fiber size with peripheral nuclei.

No lesions of significance
(52763,54807)

Head

Multiple levels through the head demonstrate dermal appendages, nasal cavity,oral cavity, teeth and tongue including muscle bundles (52891-94). Sections also show unremarkable pituitary gland including pars intermedia, pars distalis and pars nervosa
as well and the trigeminal nerve/ganglia (52890).The outer and middle regions of the ear are discernible. The tympanic membrane is intact and the ossicles are unremarkable and include the stapedial annular ligaments (52891-92).
Typical components of the inner ear including bony labyrinth, organ of corti, stria vascularis and scala cavities are discernible. Based on multiple levels, the organ of corti is unremarkable with no discernible loss of inner/outer hair cells and typical tectorial membrane (52892).
The cochlear nerve and spiral ganglion is also demonstrated and based on several levels, there is no reduction in the density of the spiral ganglion cells. Examples of otolith organs can be seen with typical features such as the hair cells and mineral otoliths. The ampulla including the crista ridge with hair cells is discernible (52892)

No lesions of significance
(52890-52894)

386

Macro Observations

Spleen: 15x5x2mm
Kidneys: 8x6x5mm, symmetrical
Thymus: 8x6x2mm
Heart: 10x8x5mm
Brain: 15x10x5mm, symmetrical
Pituitary gland identified, macroscopically normal
Tail 80mm (straight)
Teeth, tongue and oral mucosa unremarkable - Head harvested for evaluation of auditory and vestibular structures
Tail suspension test for neurological defects-negative
Left hind leg-Bone marrow smear

Ears harvested and frozen
Left tibia collected and fixed overnight in 10% NBF at room temperature then transferred to 70%

ethanol

Micro Observations

Peripheral blood smear: Examination of the smear showed red blood cells (majority of cells shown), numerous white blood cells including segmented neutrophils and platelets (clumps). No discernible morphological changes or detectable parasites (52862)

Marrow smear: Cells observed were readily identified as those from the erythroid and myeloid series. Conspicuous cells from the lymphoid series. Discernible and unremarkable megakaryoblasts (52861).

Mammary glands

Section shows typical mammary fat pad with developing lactiferous ducts (including primary duct), blood vessels and nerve
bundles. Unremarkable dermal appendages.

No lesions of significance
(52793)

Ovaries/Oviducts

Section shows unremarkable ovaries containing follicles at various stages of development and one corpus luteum.
Unremarkable oviduct micromorphology with typical columnar epithelium and mucosal folds.

No lesions of significance
(54656)

Uterus/Cervix/Vagina/Clitoral gland

Unremarkable architecture of the endometrium/ endometrial glands, myometrium and adventitia.
The endometrial stroma appeared distended judged to be estrus related. Neutrophils
and low numbers of lymphocytes identified in the endometrial glands; a common finding within a non-inflamed endometrium during the phases of estrus.
The micromorphology of the uterus and vagina places the animal at metestrus

No lesions of significance
(52792, 54656)

Urinary Bladder

Unremarkable collapsed bladder with typical urothelium and detrusor muscle

No lesions of significance
(52792, 54656)

Liver/Gall bladder

Section shows typical liver parenchyma including hepatocytes, Kupffer cells, portal triads and central veins. Unremarkable Gall bladder.

No lesions of significance
(52794)

Stomach

Section shows unremarkable fore and glandular portions of the stomach with limiting ridge.

Query mild enterocyte hyperplasia
(52801)

Comments:

Pathology to comment

Small Intestine (Duodenum, Jejunum & Ileum)/GALT

Section shows typical mucosal villi and submucosal layers. Mild enterocyte hyperplasia and prominent mitotic figures.
Peyer's patches displayed typical reactive nodal histology.
(52796, 52806)

Comments:

Pathology to comment

Cecum/Colon/GALT

Typical mucosal folds and submucosal layers and typical lymphoid clusters (Peyer's patch) with reactive micromorphology.
Marked intraluminal protozoan infestation in the cecum.
(52808, 52809)

Comments:

Pathology to comment

Mesenteric lymph node

Section shows mesenteric lymph node with typical reactive micromorphology including mild follicular hyperplasia, germinal centre formation and mild sinus histiocytosis.

No lesions of significance
(52791)

Spleen

Unremarkable follicular pattern identified with typical red and white pulp micromorphology.
Mild to moderate extramedullary hematopoiesis identified in the red pulp of the spleen.

No lesions of significance
(52804)

Pancreas

Section shows representative exocrine tissue (serous acini) and endocrine tissue (islets of Langerhans).

No lesions of significance
(52804)

Kidney

Section shows typical cortex with a uniform distribution of Glomeruli and accompanying nephron components. The micromorphology of the tubules was unremarkable.

No lesions of significance
(52795)

Adrenal glands

Section shows one adrenal gland with typical cortex/medulla micromorphology.

No lesions of significance
(52795)

Salivary glands and Regional lymph nodes

Section shows submandibular, sublingual and parotid glands.
The regional lymph nodes displayed mild follicular hyperplasia with germinal centre formation, indicating a reactive state.

No lesions of significance
(52791)

Thyroids

Normal lobes of the thyroid gland with typical colloid secreting follicles lined by cuboidal epithelium.

No lesions of significance
(52799)

Trachea/Lungs

Section shows typical lung micromorphology demonstrating parenchyma/alveoli, bronchioles and blood vessels.
Small portion of trachea with unremarkable mucosal epithelial lining and hyaline cartilage.
Oesophagus with typical features including stratified squamous epithelium

No lesions of significance
(52799)

Thymus

Section shows typical medulla/cortex distribution and micromorphology. Several small thymic cysts were identified.

No lesions of significance
(52800)

Comments:

Thymic cysts represent either a dilation of thymic tubular structures or remnants of the thymopharyngeal ducts. Cysts are a common finding in aged mice or they may be associated with epithelial glandular hyperplasia.
Pathology of the Mouse, R.R Maronpot

Pathology to comment

Heart/chambers/vessels/valves

Typical micromorphological observed in cardiac muscle, chambers, valves and vessels of the heart. The cardiac muscle fibres demonstrated typical features including central nuclei, branching fibres and striations.

No lesions of significance
(52798)

Skin

Typical dermal appendages and distribution

No lesions of significance
(52805)

Tail

Section shows typical tail components including keratinized squamous epithelium, dense regular connective tissue, tendons, bone, intervertebral disc, bone marrow, skeletal muscle, nerves and blood vessels.

No lesions of significance
(52802)

Eyes/Harderian glands

Section shows eyes with unremarkable retina, optic nerve, cornea, iris, ciliary body, lens, sclera and choroid.
Typical branched tubuloalveolar formation of the Harderian gland.

No lesions of significance
(52807)

Brain

Sections were prepared from the standard levels of the brain:

Level I: including cortex, corpus callosum, the lateral ventricles,anterior commissure and caudate putamen (Bregma -0.98)
Level II: includes the hippocampus, thalamus, amygdala,hypothalamus, lateral and third ventricles (Bregma -2.54)
Level III: includes the cerebellum, pons and fourth ventricle (Bregma -5.68)

Sections of brain stained with Haematoxylin and Eosin, Luxol Fast Blue appear symmetrical with no ventricular dilation observed, unremarkable meninges and typical lamination. The cerebellum appears symmetrical with typical architecture and Purkinje cells. At this level, there was no evidence of neuronal loss and the myelination appears normal.

No lesions of significance
(52810, 52872)

Comments:

Neuropathology to comment

Spinal cord

Representative thoracic and lumbar region of spinal cord, vertebral bone, striated muscle, peripheral nerves and bone marrow.

No lesions of significance
(52803)

(Hind leg) Long bone/Bone marrow/Synovial joint/Skeletal muscle

Levels through the hind leg show unremarkable long bone, striated muscle, synovial joint and bone marrow including conspicuous megakaryoblasts. The skeletal muscle shows consistent fiber size with peripheral nuclei.

No lesions of significance
(52797, 54809)

Head

Multiple levels through the head demonstrate dermal appendages, nasal cavity,oral cavity, teeth and tongue including muscle bundles (52901). Sections also show unremarkable pituitary gland including pars intermedia, pars distalis and pars nervosa
as well and the trigeminal nerve/ganglia (52900).The outer and middle regions of the ear are discernible. The tympanic membrane is intact and the ossicles are unremarkable and include the stapedial annular ligaments (52901-52903).

Typical components of the inner ear including bony labyrinth, organ of corti, stria vascularis and scala cavities are discernible. Based on multiple levels, the organ of corti is unremarkable with no discernible loss of inner/outer hair cells and typical tectorial membrane (52901-52903).
The cochlear nerve and spiral ganglion is also demonstrated and based on several levels, there is no reduction in the density of the spiral ganglion cells. Examples of otolith organs can be seen with typical features such as the hair cells and mineral otoliths. The ampulla including the crista ridge with hair cells is discernible (52903)

No lesions of significance
(52900-52904)

389

Macro Observations

Spleen: 12x3x2mm
Kidneys: 11x6x4mm, symmetrical
Thymus: 9x9x2mm
Heart: 8x6x6mm
Brain: 15x10x6mm, symmetrical
Pituitary gland identified, macroscopically normal
Tail 80mm (straight)
Teeth, tongue and oral mucosa unremarkable - Head harvested for evaluation of auditory and vestibular structures
Tail suspension test for neurological defects-negative
Left hind leg-Bone marrow smear

Ears harvested and frozen
Left tibia collected and fixed overnight in 10% NBF at room temperature then transferred to 70% ethanol

Micro Observations

Peripheral blood smear: Examination of the smear showed red blood cells (majority of cells shown), occasional white blood cells including segmented neutrophils and platelets (clumps). No discernible morphological changes or detectable parasites (52866)

Marrow smear: Cells observed were readily identified as those from the erythroid and myeloid series. Conspicuous cells from the lymphoid series. Discernible and unremarkable megakaryoblasts (52865).

Mammary glands

Section shows typical mammary fat pad with developing lactiferous ducts, blood vessels, nerve bundles and a large reactive lymph node. Some scattered mast cells identified; a common finding in mouse mammary tissue.

No lesions of significance
(52836)

Ovaries/Oviducts

Section shows unremarkable ovaries containing follicles at various stages of development and several corpora lutea.
Unremarkable oviduct micromorphology with typical columnar epithelium and mucosal folds.

No lesions of significance
(52843)

Uterus/Cervix/Vagina/Clitoral gland

Unremarkable architecture of the endometrium/ endometrial glands, myometrium and adventitia. Some neutrophils and low numbers of lymphocytes identified in the endometrial glands; a common finding within a non-inflamed endometrium during the phases of estrus. The micromorphology of the uterus and vagina places the animal at metestrus.

No lesions of significance
(52843)

Urinary Bladder

Section does not include bladder

Liver/Gall bladder

Section shows typical liver parenchyma including hepatocytes, Kupffer cells, portal triads and central veins. Unremarkable Gall bladder

No lesions of significance
(52838)

Stomach

Section shows unremarkable fore and glandular portions of the stomach with limiting ridge.
Query mild enterocyte hyperplasia
(52846)

Comments:

Pathology to comment

Small Intestine (Duodenum, Jejunum & Ileum)/GALT

Section shows typical mucosal villi and submucosal layers. Mild enterocyte hyperplasia and prominent mitotic figures.
Peyer’s patches displayed typical reactive nodal histology.
(52834, 52850)

Cecum/Colon/GALT

Typical mucosal folds and submucosal layers and typical lymphoid clusters (Peyer's patch) with reactive micromorphology.
Marked intraluminal protozoan infestation in the cecum and proximal colon.
(52831-32)

Comments:

Pathology to comment

Mesenteric lymph node

Section shows typical reactive mesenteric lymph node with representative cortex including the occasional follicle, an expansive paracortical area and mild sinus histiocytosis.

No lesions of significance
(52840)

Spleen

Unremarkable follicular pattern identified with typical red and white pulp micromorphology.
Mild extramedullary hematopoiesis identified in the red pulp of the spleen, a common finding in the mouse.

No lesions of significance
(52837)

Pancreas

Section shows representative exocrine tissue (serous acini) and endocrine tissue (islets of Langerhans).
No lesions of significance
(52837)

Kidney

Section shows a cortex and papilla. There is a uniform distribution of Glomeruli and accompanying nephron components and the micromorphology of the convoluted and straight segment tubules was unremarkable.

No lesions of significance
(52848)

Adrenal glands

Section shows one adrenal gland with typical cortex/medulla micromorphology.

No lesions of significance
(52848)

Salivary glands and Regional lymph nodes

Section shows submandibular, sublingual and parotid glands.
The regional lymph nodes displayed mild follicular hyperplasia with germinal centre formation, indicating a reactive state.

No lesions of significance
(52840)

Thyroids

Section does not include thyroid glands

Trachea/Lungs

Section shows typical lung micromorphology demonstrating parenchyma/alveoli, bronchioles, blood vessels and parabronchial lymph node. Various degrees of parenchymal congestion and collapse judged to be artefactual.
Trachea with unremarkable mucosal epithelial lining and hyaline cartilage
Oesophagus with typical features including stratified squamous epithelium

No lesions of significance
(52849)

Thymus

Section shows typical medulla/cortex distribution and micromorphology.

No lesions of significance
(52844)

Heart/chambers/vessels/valves

Typical micromorphological observed in cardiac muscle, chambers, valves and vessels of the heart. The cardiac muscle fibres demonstrated typical features including central nuclei, branching fibres and striations.

No lesions of significance
(52847)

Skin

Typical dermal appendages and distribution

No lesions of significance
(52839)

Tail

Section shows typical tail components including keratinized squamous epithelium, dense regular connective tissue, tendons, bone, bone marrow, skeletal muscle, nerves and blood vessels.

No lesions of significance
(52841)

Eyes/Harderian glands

Section shows eyes with representative retina, optic nerve, cornea, iris, ciliary body, lens, sclera and choroid.
Typical branched tubuloalveolar formation of the Harderian gland.

No lesions of significance
(52833)

Brain

Sections were prepared from the standard levels of the brain:

Level I: including cortex, corpus callosum, the third and lateral ventricles, choroid plexus and caudate putamen (Bregma -1.58)
Level II: includes the hippocampus, thalamus, amygdala,hypothalamus, lateral and third ventricles (Bregma -1.82)
Level III: includes the cerebellum, pons and fourth ventricle (Bregma -5.68)

Sections of brain stained with Haematoxylin and Eosin, Luxol Fast Blue appear symmetrical with no ventricular dilation observed, unremarkable meninges and typical lamination. The cerebellum appears symmetrical with typical architecture and Purkinje cells. At this level, there was no evidence of neuronal loss and the myelination appeared normal.

No lesions of significance
(52835, 52874)

Comments:

Neuropathology to comment

Spinal cord

Representative thoracic and lumbar region of spinal cord, vertebral bone, striated muscle,peripheral nerves and bone marrow.

No lesions of significance
(52842)

Comments:

Neuropathology to comment

(Hind leg) Long bone/Bone marrow/Synovial joint/Skeletal muscle

Levels through the hind leg show unremarkable long bone, striated muscle, synovial joint and bone marrow including conspicuous megakaryoblasts. The skeletal muscle shows consistent fiber size with peripheral nuclei.

No lesions of significance
(52845, 54811)

Head

Multiple levels through the head demonstrate dermal appendages, nasal cavity,oral cavity, teeth and tongue including muscle bundles (52910-11). Sections also show unremarkable pituitary gland including pars intermedia, pars distalis and pars nervosa
as well and the trigeminal nerve/ganglia (52910).The outer and middle regions of the ear are discernible. The tympanic membrane is intact and the ossicles are unremarkable and include the stapedial annular ligaments (52911-12).
Typical components of the inner ear including bony labyrinth, organ of corti, stria vascularis and scala cavities are discernible. Based on multiple levels, the organ of corti is unremarkable with no discernible loss of inner/outer hair cells and typical tectorial membrane (52912).
The cochlear nerve and spiral ganglion is also demonstrated and based on several levels, there is no reduction in the density of the spiral ganglion cells. Examples of otolith organs can be seen with typical features such as the hair cells and mineral otoliths. The ampulla including the crista ridge with hair cells is discernible (52913)

No lesions of significance
(52910-14)

Comment / Plan

Case APN16/010 will be referred to Professor Rolfe Howlett R&A Pathology Services NSW for a supplementary pathology report and Dr. John Finnie-SA Pathology for a neuropathology report.

Tina Cardamone
19th April, 2016

Supplementary Pathology Report

380 (control)

388 (control)

378

Peripheral blood smear:
The smear displayed neutrophils and lymphocytes being the main white cell types; the most numerous cell type being the erythrocyte

Marrow smear:
Myeloid and erythroid cells were readily identified throughout which were conspicuous darkly stained small cells. The latter cells were presumed to be a mixture of normocytes and small lymphoid cells.
A heavy sprinkling of large elongated microbiological bacterial like micro-organisms occurred through out the marrow cells.
These microorganisms were cigar shaped and their length measured 1 to 2 the diameter of a red blood cell. As none of the other mice displayed these micro-organism in their marrow smears their presence is judged to be a contaminant.

Testes and Epididymes

There are no abnormalities in the testes, epididymes and vas deferens. Intraluminal storage of spermatozoa was observed in the tail of epididymis and the vas deferens.
No lesions of significance

Secondary sex glands (e.g. seminal vesicles; prostate, coagulating gland)

Unremarkable seminal vesicles, prostate- and coagulating- glands
A large concretion in the prosthetic urethra appeared to have caused prominent dilation of the urethral segment; such concretions are often observed as co-incidental findings.
No lesions of significance

Prepuce ,Penis, Preputial gland, Urethra

The sections display these structures clearly and no lesions of significance were observed.

Urinary Bladder

The bladder was sectioned in slide No.52691 and the features displayed a distended bladder wall lined with urothelium; there were no lesions of significance

Liver /Gall Bladder

Sections display a large section of liver and its gall bladder.
No lesions of significance

Oesophagus

A longitudinal section of the oesophagus from the thoracic mediastinum did not display any features of significance(barcode 52690)

Stomach

Section displays the glandular and non-glandular regions of the stomach separated by the limiting ridge
No lesions of significance

Small Intestine

The sections display well formed epithelial lining with well formed villi enclosed by normal musculature coats. In a few local regions with epithelial tips of the villi being unstained and ballooning, this is judged to be artefact. The epithelium could be considered as mildly hyperplastic but without appropriately prepared sections this apparent hyperplasia is probably within normal limits.
The Peyer's patches showed mild reactivity; a constant observed feature in the small bowel.
No lesions of significance

Large Intestine /Caecum

Sections from the caecum and the colon displayed numerous intraluminal protozoa
These micro-organism had not entered and parasitized the colonic epithelial lining.
Hence they are best considered non- pathogenic.
No lesions of significance

Mesenteric Lymph Node

Mild follicular hyperplasia and mild sinus histiocytosis
The mesenteric lymph node frequently shows these changes; as would be expected because these nodes drain the small intestine.
No lesions of significance

Spleen

The microscopic pattern of relatively discreet white cell islands scattered throughout the splenic red cell parenchyma failed to show any lesion of significance.
A common finding extramedullary haematopoiesis was observed; such is almost considered normal.
No lesions of significance

Pancreas

The parenchyma of this organ displayed endocrine (Islets of Langerhans) and exocrine tissue. In a normal and expected pattern
No lesions of significance

Kidneys

In the distal convoluted tubular region in both kidneys small amounts of protein appear to have collected within the lumens to form hyaline casts. In a few instances this protein appears about the size of an epithelial cell.
The glomerulae were scattered evenly throughout the cortex. No abnormal features were observed within the glomeruli .

Adrenal glands

Both adrenals were included in the sections and reveal no lesion of significance

Thyroid glands

I concur with the features described in the preliminary report
No lesions of significance

Salivary glands and associated Lymph nodes

All three glands are present in the section . There does not appear to be any lesion of significance in any of the three gland types; Parotid, Sublingual and Submandibular.
No lesions of significance

Lungs/ Trachea /Bronchi

Sections include both lungs and airways including the trachea with its cartilaginous support.
No lesions of significance

Thymus

The microscopic features of cortex and medulla of this gland were within the expected cellular parameters.
No lesions of significance

Heart / major vessels / valves

Left and right ventricles of the cardiac organ seemed normal as does the pulmonary artery and left and right atria. Myocardial cells seem appropriate and within normal parameters
No lesions of significance

Skin

Normal epidermis, dermis and dermal appendages as well as hypodermis and Panniculus carnosus..
The follicles appear evenly spaced.
No lesions of significance

Eyes / Harderian glands

Sections display both eyes appear to contain all structures mentioned in the preliminary report.
Tubuloalveolar Harderian glands have no lesion of abnormality.
No lesions of significance

Spinal Cord/Vertebrae

Representative sections from the thoracic and lumbar regions (3 transverse and one longitudinal section) appeared not to display any feature of significance
No lesions of significance

Skeletal Tissue(appendicular skeleton)/ synovial joint/ skeletal muscle

Sections from 2 levels reveal the distal femur and proximal tibia with the interposing styfle joint as well as adjoining normal skeletal muscle. Some bones from the tarsus and foot were present and appear normal.
No lesions of significance

Tail

I concur with the preliminary report and its detail.
No lesions of significance

Head

The preliminary report details the observed microscopic features of the oral and nasal cavities as well as the pituitary gland.
Moreover the organ of Corti and other auditory components, including the outer, middle and inner ear as well as those involved in balance were identified and details were included.
No lesions of significance

381

Blood smear:
Concur with your remarks

Marrow smear:
Cells from myeloid and erythroid series were present
The deeply and over-stained small cells are probably a mixture of normocytes and small lymphocytes. Examination of the marrow in skeletal sections showed no excessive lymphoid cells.

Ovary/ Fallopian tubes

I concur with the preliminary notes; no lesions of significance

Uterus / Vagina

I concur with the preliminary notes; no lesions of significance

Mammary glands

I concur with the preliminary notes; no lesions of significance

Urinary Bladder

I concur with the preliminary notes; no lesions of significance

Liver /Gall Bladder

I concur with the preliminary notes but would add there was a small focus of neutrophils in the parenchyma with a concomitant of hepatocytic loss; this feature is often observed in rodent livers and is of no significance.
No lesions of significance

Oesophagus

No lesions of significance in the section cut from the thoracic portion.

Stomach

I concur with the preliminary notes to which I would add there was a mild superficial gastritis in the pyloric mucosa just proximal to the pyloric sphincter.
No lesions of significance

Small Intestine

In my opinion changes in the mucosal depth from sections prepared in such a manner, in order to examine large areas of the intestinal mucosa, are not appropriate to judge mild degrees of hyperplasia because of the variation from animal to animal.
No lesions of significance

Large Intestine /Caecum

Prominent intraluminal protozoan infestation. There did not appear to be invasion of these microbiological agents into the mucosal walls.
Tritrichomonas muris and T. minuta are well known parasites that are commensal in mice.
I concur with the preliminary notes; no lesions of significance

Spleen

I concur with the preliminary notes; no lesions of significance

Pancreas

I concur with the preliminary notes; no lesions of significance

Kidneys

I concur with the preliminary notes; no lesions of significance

Adrenal glands

I concur with the preliminary notes; no lesions of significance

Mesenteric Lymph Node

I concur with the preliminary notes; no lesions of significance

Thyroid glands

I concur with the preliminary notes; no lesions of significance

Salivary glands and associated Lymph nodes

I concur with the preliminary notes; no lesions of significance

Lungs/ Trachea /Bronchi

I concur with the preliminary notes; no lesions of significance

Thymus

I concur with the preliminary notes; no lesions of significance

Heart / major vessels / valves

I concur with the preliminary notes; no lesions of significance

Skin

I concur with the preliminary notes; no lesions of significance

Eyes / Harderian glands

I concur with the preliminary notes; no lesions of significance

Skeletal Tissue(appendicular skeleton)/ synovial joint/ skeletal muscle

I concur with the preliminary notes; no lesions of significance in these tissues

Head

Four levels were cut and features listed in the preliminary report was detailed and instructive. No lesions of significance were observed in the pituitary gland or nasal and oral cavities or in the hearing and balance organelles

Spinal Cord

I concur with the preliminary notes; no lesions of significance

Tail

I concur with details presented in the preliminary report. No lesions of significance

383

Peripheral blood smear:
The smear displayed neutrophils and lymphocytes being the main white cell types; the most numerous cell type being the erythrocyte

Marrow smear:
Myeloid and erythroid cells were readily identified. Darkly stained small cells were conspicuous.

Testes Epididymis vas Deferens

Typical convoluted seminiferous tubular architecture with no lesions in both testes.
The epididymis from both testis stores the spermatids in the tail of the epididymis.
No lesions of significance

Secondary sex glands (e.g. seminal vesicles; prostate, coagulating gland)

The features described in the preliminary report are as detailed.
There are no lesions of significance in the seminal vesicles, prostate or the coagulating gland
Some concretion in the prostatic urethra with birefringent crystals
No lesions of significance

Penis, preputial gland

No lesions of significance

Urinary Bladder

No lesions of significance
The urothelium was stretched

Liver /Gall Bladder

I concur with the observations in the preliminary report
There are no lesions of significance

Oesophagus and Stomach

In the lumens of these organs were protozoan micro-organisms; such supports coprophagia.
No lesions of significance

Large Intestine /Caecum

Intraluminal protozoan micro-organisms were packed into the lumen. There was no evidence of the protozoan invading or living in the mucosa.
Coprophagy would enhance the proliferation of these non-parasitic micro-organisms.

Small Intestine

The issue of mild epithelial hyperplasia is difficult to assess. In this particular intestine there are areas where the villous tips are oedematous and in some regions the villi are necrotic (artefact). Additionally this mouse was recorded as having mildly distended bowel.

Mesenteric Lymph Node

A small amount of node displayed minimal changes
No lesions of significance

Spleen

The parenchyma of the spleen has a pattern that displays no lesions of significance
Extramedullary haematopoiesis is commonly found in normal mice. See the additional notes provided from the preliminary report

Pancreas

The features of the endocrine glands(Islets of Langerhans) and the exocrine glands
Have no lesions of significance

Kidneys

Sections of both kidneys display no lesions of significance

Adrenal glands

Sections of only one adrenal was provided. There were no lesions of significance in the cortex or medulla.

Thyroid glands

I concur with the features noted in the preliminary report
There are no lesions of significance

Salivary glands and associated Lymph nodes

The three glands were sampled and show no abnormalities. The regional lymph nodes display a mild sinus histiocytosis ; such reactivity is frequently observed in these lymph glands.
No lesions of significance

Lungs/ Trachea /Bronchi

I concur with the features noted in the preliminary report
There are no lesions of significance

Thymus

I concur with the features noted in the preliminary report
There are no lesions of significance

Heart / major vessels / valves

The four chambers , the atria-ventricular valves,
aorta and pulmonary artery were identified. No lesions of significance

Skin

I concur with the features noted in the preliminary report
There are no lesions of significance

Eyes / Harderian glands

Features regarding the eyes and the Harderian glands were unremarkable.
No lesions of significance

Spinal Cord /vertebrae

I concur with the features noted in the preliminary report
There are no lesions of significance

Skeletal Tissue(appendicular skeleton)/ synovial joint/ skeletal muscle

Sections from 2 levels reveal normal features of bones, striated muscle Styfle joint and bone marrow.
No lesions of significance

Tail

The components of the tail e.g. skin, subcutaneous tissue , muscle, coccygeal bone, intervertebral discs show no abnormal features.
No lesions of significance

Head

Four levels were cut and revealed no abnormalities in the pituitary gland or the oral and nasal cavities. The levels displayed showed the outer and middle regions of the ear as well as the components of the inner ear.
The detailed preliminary report is excellent and multiple sections failed to display any lesion of significance

385

Peripheral blood smear:
Majority of blood cells were erythrocytes but where the smear preparation was thin the most common cell types were lymphocytes and segmented neutrophils

Marrow Smear:
Erythroid and myeloid cells were identified readily. One of the common cell types present and readily recognized but unidentified were small darkly stained cells; the most likely cells are normocytes and/or small lymphocytes

Testes, Epididymis, vas Deferens

The testicular tubular structure is without significant lesions. Intraluminal spermatozoa were encountered within the tail of the epididymis.
No lesions of significance

Secondary sex glands (e.g. seminal vesicles; prostate, coagulating gland)

Seminal vesicles, prostate glands and coagulating glands appear not to have any lesion of significance

Penis, preputial gland, urethra

The penis and prepuce and urethra as well as preputial glands appear not to have any lesion of significance

Urinary Bladder

The bladder wall and its components are distended but with no lesion of significance

Liver /Gall Bladder

The normal components of this organ do not display any feature of abnormality.
There was no gall bladder in the section.
No lesion of significance

Oesophagus

A portion of the oesophagus was present with the section of the liver
A few bacterial colonies had colonized the keratinous surface.
A longitudinal section of the mediastinal oesophagus was normal showing no abnormalities.
No lesion of significance

Stomach

The glandular and non-glandular regions were separated by the limiting ridge . The glandular portion of the stomach at the pyloric sphincter may appear mildly hyperplastic but this is artefactual.
No lesions of significance

Small Intestine

The mucosal epithelial lining with villi is viable and somewhat dominates the lumen.
No lesion of significance

Large Intestine /Caecum

Protozoa fill the lumen of the large intestine but do not invade the mucosa.
No lesions of significance

Mesenteric Lymph Node

A mild follicular hyperplasia
No lesions of significance

Spleen

The spleen displays a normal pattern of microscopic detail
Secondary haematopoiesis is frequently observed in most murine strains.
No lesions of significance

Pancreas

The endocrine and exocrine portions of the pancreas appears within normal parameters microscopically
No lesions of significance

Kidneys

Components of the kidney including the glomeruli, nephrons forming the cortex and the medulla and the renal pelvis show no feature of significant abnormality. However a few distal convoluted tubules contain hyaline casts. There was no reflux changes in the glomeruli of proximal tubular lumens.
The marked patchy congestion appears artefactual.

Adrenal glands

One adrenal was sectioned and the cortex and small portion of the medulla was present, no changes of significance

Thyroid glands

No lesions of significance

Salivary glands and associated Lymph nodes

All three glands were represented.
The draining lymph nodes displayed a mild follicular hyperplasia
No lesion of significance

Lungs/ Trachea /Bronchi

Sections included the left and right lungs together with the extra pulmonary
Trachea with a para tracheal lymph node.
No lesions of significance

Thymus

Typical cortex and medulla with a small solitary cyst. The preliminary report included a comment from a reference edited by R.R. Maronpot

Heart / major vessels / valves

Sections display left and right ventricles and portions of the left and right atria-ventricular valves.
Three of the valves had a leading edge displaying myxomatous thickening.
The significance cant be judged until more advanced ages

Skin

The skin had normal epidermis, dermis and follicular distribution
The dermal appendages are normal as are the upper hypodermis and muscular Panniculus carnosus

Eyes / Harderian glands

I concur with the preliminary report
No lesions of significance

Skeletal Tissue(appendicular skeleton)/ synovial joint/ skeletal muscle

Sections reveal the distal femur, and proximal tibia. The joint has not been opened(section not deep enough) but enclosing tissue seems normal. The skeletal muscle appears normal as does the cellular content within the bone marrow.

Head

I concur with the careful documentation of all the features exposed in the sections cut at the 4 levels.
No lesions of significance

Tail

The revealed components in cross and longitudinal sections are normal. The preliminary report lists all those components
No lesions of significance

Spinal Cord /vertebrae

Representative sections from the thoracic and lumbar regions appeared not display any feature of significance
No lesions of significance

386

Peripheral blood smear:
Majority of blood cells were erythrocytes but where the smear preparation was good the most common cell types were lymphocytes and segmental neutrophils

Marrow Smear:
Erythroid and myeloid cells were identified readily. One of the common cell types readily recognized were small darkly stained cells that were most likely a mixture of normocytes and lymphocytes

Ovary/ Fallopian tubes

Follicles at varying stages that were unremarkable
Fallopian tubules with no significant abnormalities

Uterus / Vagina

No lesions of significance. In the deeper section the vaginal features of a heavy layer of keratin together with considerable inflammatory cells does suggest that mouse was leaving the oestrus phase but yet to show the characteristic pattern of metoestrus.

Mammary glands

I concur with details presented in the preliminary report. No lesions of significance

Urinary Bladder

I concur with details presented in the preliminary report. No lesions of significance

Liver /Gall Bladder

I concur with details presented in the preliminary report. No lesions of significance

Oesophagus

The section of the thoracic portion of the oesophagus does not display any lesions of significance

Stomach

There are no features of significance in the sections prepared from the stomach. The limiting ridge divides the lining of this organ into glandular and non-glandular regions.
Whether areas of the glandular segment is mildly hyperplastic is debatable.

Small Intestine

No lesions of significance
The villi of the mucosa do appear slightly longer but seem to be within expected parameters.
Indeed the features of the mucosa of this animal seem similar to the control female mouse.

Large Intestine /Caecum

Marked protozoan intraluminal accumulation in the caecum; note the protozoan organism are not within the mucosa. Common non pathogenic protozoa listed are Tritrichomonas muris and others
Intra-lumenal protozoan organisms are observed in small numbers in the colon.

Mesenteric Lymph Node

Moderate follicular hyperplasia and sinus histiocytosis
The mesenteric lymph node is a perpetual state of reactivity.

Spleen

I concur with details presented in the preliminary report. No lesions of significance

Pancreas

I concur with details presented in the preliminary report. No lesions of significance

Kidneys

I concur with details presented in the preliminary report. No lesions of significance

Adrenal glands

Only one adrenal was sectioned.
No lesions of significance in the cortex or medulla of this adrenal

Thyroid glands

I concur with details presented in the preliminary report.
No lesions of significance

Salivary glands and associated Lymph nodes

No lesions in the 3 salivary glands (sublingual, parotid and submandibular)
In the para-glandular lymph nodes, mild follicular hyperplasia and sinus histiocytosis.
No lesions of significance.

Lungs/ Trachea /Bronchi

I concur with details presented in the preliminary report. No lesions of significance

Thymus

The medulla and cortex of the thymus are without significant lesions. There were several small cysts and these occur regularly in mice.
No lesions of significance
The comment re thymic cysts in the preliminary report from Maronpot is appropriate.

Heart / major vessels / valves

I concur with the details in the preliminary report to which I would add the following:
There is a mild myxomatous thickening of 2 leaflets of the left atria-ventricular valve.
The latter changes are probably of minimum significance

Skin

The epidermis, dermis upper hypodermis and Panniculus carnosus together with Dermal appendages do display any features of significance.

Eyes / Harderian glands

One eye appeared smaller than the other but this would appear to be due to plane of section (towards the ocular margins); particularly as all components normally found in the eye were present.
No features of significance

Spinal Cord /vertebrae (3 sections of the spinal cord from the thoracic and lumbar regions)

I concur with the preliminary report findings.
No lesions of significance

Skeletal Tissue(appendicular skeleton)/ synovial joint/ skeletal muscle

The section was prepared from the hind limb. No lesions occur in the bones, styfle joint and striated muscle. The bone marrow had conspicuous megakaryocytes but other wise seemed to have cells from myeloid and erythroid series

Tail

I concur with details presented in the preliminary report. No lesions of significance

Head

I concur with the detailed preliminary report indicating that the nasal and oral cavities had no significant lesions. Furthermore, microscopic features of auditory and balance were identified and seemed not to display any abnormal features.
No significant lesions.

389

Blood smear:
Occasional white blood cell, (often small with little in the way of cytoplasm) in a dominant background of red blood cells.

Marrow smear:
Erythroid and myeloid cells were readily identified. A commonly identified cell type was small densely stained nucleated cell; the most likely types being a normocytes and lymphocytes.

The marrow smear and sections cut from the trabeculated bone do suggest that there is a possible increase in lymphocytes in the marrow.

Ovary/ Fallopian tubes

One ovary displaying follicles at varying stages together with several corpora lutea.
The fallopian tubules were lined by intact columnar epithelium
No lesion of significance

Uterus / Vagina

The microscopic features of uterus ,myometrium, endometrial glands with mild numbers of migrating neutrophils in the uterine walls suggests that this animal was in metoestrus.
No lesions of significance.

Mammary glands

I concur with the preliminary notes. No significant changes

Urinary Bladder

A focus of neutrophils and loss of hepatocytes in the hepatic parenchyma(size is 0.01mm)
These tiny lesions are routinely observed in murine hepatic sections
No lesions of significance.

Oesophagus

No significant lesion in the thoracic portion

Stomach

Sections show a fore and glandular portion of the stomach lining separated by a limiting ridge.
I don't think the pyloric mucosal is hyperplastic.
No lesions of significance

Small Intestine

I concur with the preliminary notes; the possibility of mild hyperplasia does exist but proof lies within appropriate multiple transverse sections rather than longitudinal as in the preparation in this case.
Two small calcific foci in the serosal layer.
No lesions of significance.

Large Intestine /Caecum

A heavy protozoan population exists with the large bowel lumen. To note the micro-organism has not populated the mucosal lining of the caecum or the colon
No lesions of significance

Mesenteric Lymph Node

I concur with the preliminary notes and there is no lesion of significance

Spleen

I concur with the preliminary notes and there is no lesion of significance

Pancreas

I concur with the preliminary notes and there is no lesion of significance

Kidneys

Both kidneys are present.
No lesions of significance occur.

Adrenal glands

The section contained a whole adrenal gland as well as a portion of the other adrenal cortex
No lesions of significance

Salivary glands and associated Lymph nodes

I concur with the preliminary notes and there is no lesion of significance

Lungs/ Trachea /Bronchi

Sections reveal pulmonary parenchyma and airways for both lungs.
The trachea with mucosa leading into the intra lobular bronchi and bronchioles.
No lesions of significance

Thymus

No lesion of significance

Heart / major vessels / valves

The 4 chambers were present. A mesenchymal valvular nodule on a leaflet of the left atria-ventricular valve.
The significance is difficult to judge.

Skin

Sections reveal normal epidermis and dermis with the dermal appendages distributed and separated in a regular manner.
No lesions of significance

Eyes / Harderian glands

Both eyes were normal with all features of retina, cornea, iris, ciliary body, lens, sclera and choroid
No lesions of significance
The tubuloalveolar formation of the Harderian glands appear to have no significant lesions.

Skeletal Tissue(appendicular skeleton)/ synovial joint/ skeletal muscle

No lesions of significance in the bones, styfle joint and striated muscle of the limb.

Head

Multiple levels(4) were examined and the features as delineated in the detailed preliminary report were accurate and with which I concur. There were no lesions of significance in the external, middle and inner ear and nasal and oral cavities.
Micromorphologically the pituitary gland appeared as expected with no lesions of significance.

Spinal cord

I concur with the preliminary report findings.
No lesions of significance

Summary

RENAL OBSERVATION / COMMENT:
In the following kidneys small numbers of tubular casts were observed in the distal convoluted tubules in at least one kidney from each of the following mice; Mouse Numbers 378; 385 as well as the control mice 380 and 388.
The presence of tubular proteinaceous casts should be further explored. Such an undertaking is worth while if this is a new strain of mouse, because there is a possibility that this strain of mouse is carrying a renal defect.

BONE MARROW SMEAR OBSERVATION/COMMENT:
The preliminary reports for bone marrow identified conspicuous lymphoid series in all the mice submitted. Indeed, considerable numbers of small deeply staining cells were present in all samples I examined. I presumed that these cells were either normoblasts/normocytes or small lymphocytes.

2nd May, 2016

Supplementary Neuropathology Report

380 (control)

H&E and LFB stained brain sections were examined. Representative
H&E stained sections of spinal cord were examined.
Slides were used as histological controls

388 (control)

H&E and LFB stained brain sections were examined. Representative
H&E stained sections of spinal cord were examined.
Slides were used as histological controls

378

H&E and LFB stained brain sections were examined. Representative
H&E stained sections of spinal cord were examined.
Sections of brain and spinal cord show no significant neuropathology.

381

H&E and LFB stained brain sections were examined. Representative
H&E stained sections of spinal cord were examined.
Sections of brain and spinal cord show no significant neuropathology.

383

H&E and LFB stained brain sections were examined. Representative
H&E stained sections of spinal cord were examined.
Sections of brain and spinal cord show no significant neuropathology.

385

H&E and LFB stained brain sections were examined. Representative H&E stained sections of spinal cord were examined.
Sections of brain and spinal cord show no significant neuropathology.

386

H&E and LFB stained brain sections were examined. Representative H&E stained sections of spinal cord were examined.
Sections of brain and spinal cord show no significant neuropathology.

389

H&E and LFB stained brain sections were examined. Representative H&E stained sections of spinal cord were examined.
Sections of brain and spinal cord show no significant neuropathology.

Summary

Sections of brain and spinal cord show no significant neuropathology.

6th May, 2016

The Australian Phenomics Network advises all research groups that images or results obtained through the services offered by the APN are to be acknowledged in resultant publications. Example acknowledgement: "This study utilised the Australian Phenomics Network Histopathology and Organ Pathology Service, University of Melbourne."

Figure S1 (relate to Figure 4). Heraud-Farlow et al

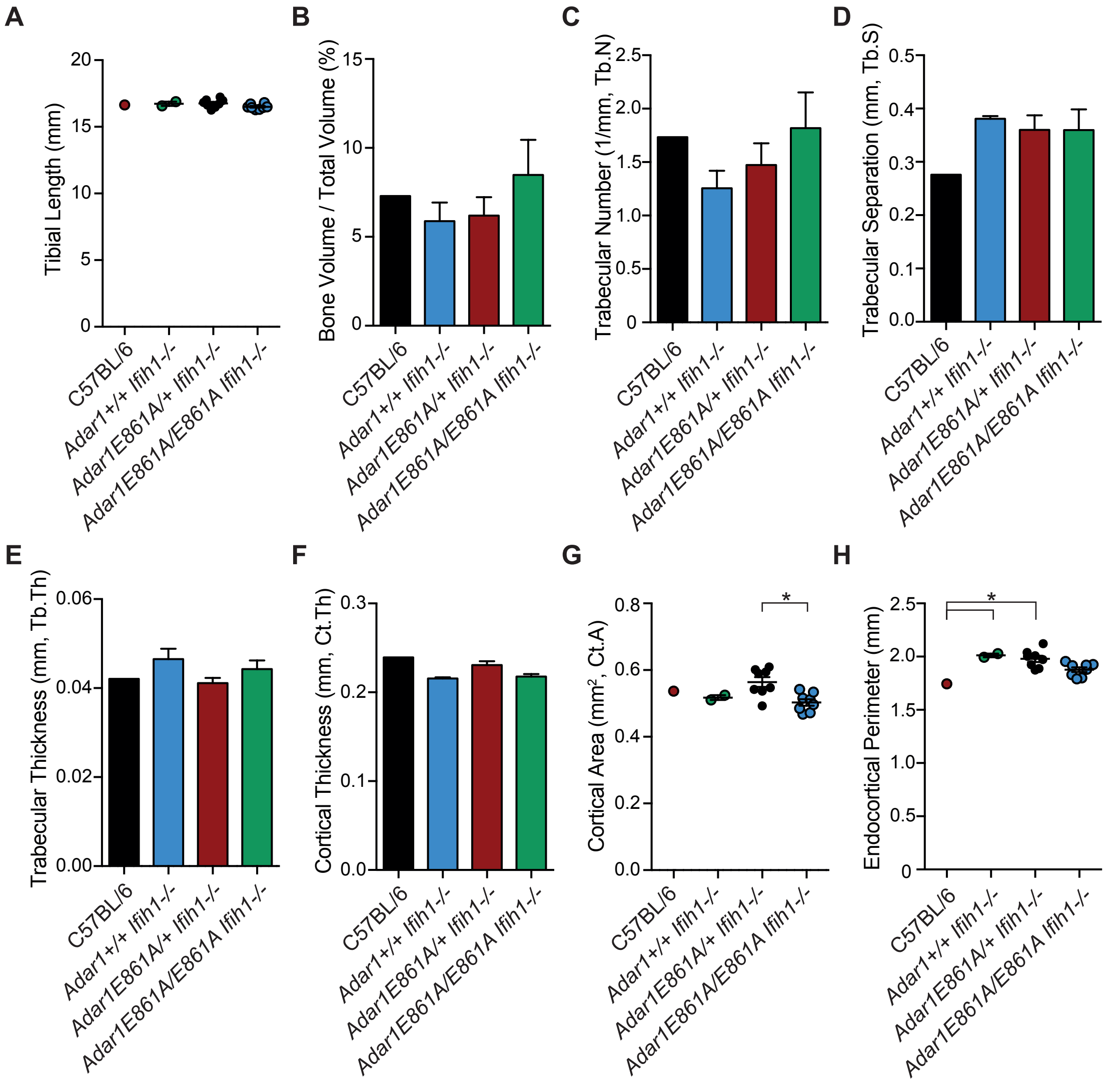

Additional File 3: Figure S1.pdf (related to Figure 4) – 12-week old female mouse bone parameters

MicroCT analysis of tibial bone parameters for the indicated genotypes from 12-week old female mice. C57BL/6 (n=1), *Adar1*$^{+/+}$*Ifih1*$^{-/-}$ (n=2), *Adar1*$^{E861A/+}$*Ifih1*$^{-/-}$ (n=8), *Adar1*$^{E861A/E861A}$*Ifih1*$^{-/-}$ (n=8). (**A**) Tibial length (mm), (**B**) Trabecular bone volume (BV/TV), (**C**) trabecular number (Tb.N), (**D**) trabecular separation (Tb.Sp), and (**E**) trabecular thickness (Tb.Th). (**F-H**) MicroCT analysis of cortical bone parametres for the same samples used for panels A-E. (**F**) Cortical thickness (Ct.Th), (**G**) cortical bone area (Ct.Ar), and (**H**) endocortical perimeter (mm). Results are graphed as mean±SEM; Statistical significance was tested using a one-way ANOVA, where *P<0.05.

Figure S2 - related to figure 7 Heraud-Farlow et al

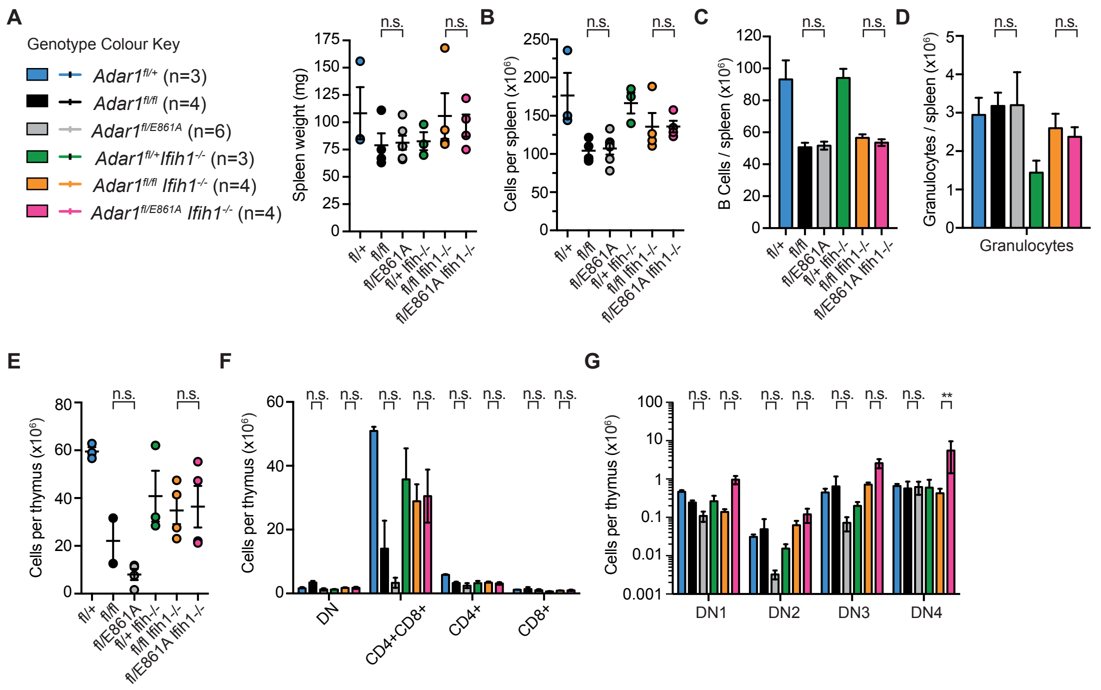

Additional File 3: Figure S2.pdf (related to Figure 7) – Spleen and thymus data from *R26*-CreER *Adar1*$^{fl/fl}$*Ifih1*$^{-/-}$ and *R26*-CreER *Adar1*$^{fl/E861A}$*Ifih1*$^{-/-}$ and control animals.

Mice of the indicated genotypes were treated with tamoxifen as described in Figure 7. Spleen (**A**) weight, (**B**) cellularity, (**C**) B cells per spleen and (**D**) granulocytes per spleen. Thymus (**E**) cellularity, (**F**) CD4/CD8 composition (DN, double negative) and (**G**) thymic progenitors. Number of animals per group as indicated in panel 7B. Results are graphed as mean±SEM; *P<0.05; **P<0.01, ***P<0.001.

Additional file 3: Table S2

Supplemental Table 2: Primers used in this study

Primer Name	Primer sequence (5'→ 3')
mIfi44 qPCR f1	CCCCTGCCATTTATTCTGTGT
mIfi44 qPCR r1	CGGATGGTTTGATGTGATTGG
mRsad2 qPCR f1	ACACAGCCAAGACATCCTTC
mRsad2 qPCR r1	CAAGTATTCACCCCTGTCCTG
mCcl2 qPCR f1	GTCCCTGTCATGCTTCTGG
mCcl2 qPCR r1	GCTCTCCAGCCTACTCATTG
mCxcl10 qPCR f1	TCAGCACCATGAACCCAAG
mCxcl10 qPCR r1	CTATGGCCCTCATTCTCACTG
mPPIA qPCR f2	GTC AAC CCC ACC GTG TTC TT
mPPIA qPCR r2	CTG CTG TCT TTG GAA CT TTG
Mm ADAR1 qPCR Set2 FOR-JBL	GAC CAG AGA TGG GAA AGC AT
Mm ADAR1 qPCR Set2 REV-JBL	CAA CTC TTT CCC TGG TCC AT
Mm ADAR2 qPCR FOR-JBL	TGT AAG CAC GCG CTG TAC TGT
Mm ADAR2 qPCR REV-JBL	GAC TCG TGG TAT GTG GTA GGC TTA G
Adar3 qPCR F2	ACCACAAACACAGAGGATGAAG
Adar3 qPCR R2	CCTTTTCCTTTTAACGCTGGG
